# More than Meets the ISG15: Emerging Roles in the DNA Damage Response and Beyond

**DOI:** 10.3390/biom10111557

**Published:** 2020-11-15

**Authors:** Zac Sandy, Isabelle Cristine da Costa, Christine K. Schmidt

**Affiliations:** Manchester Cancer Research Centre, Division of Cancer Sciences, School of Medical Sciences, Faculty of Biology, Medicine and Health, University of Manchester, Manchester M20 4GJ, UK; zac.sandy@postgrad.manchester.ac.uk (Z.S.); isabellecristine.dacosta@manchester.ac.uk (I.C.d.C.)

**Keywords:** ubiquitin-like protein (UBL), ISG15 and ISGylation, UBA7 (UBEL1), UBE2L6 (UBCH8), EFP (TRIM25) and HERC5, USP18 (UBP43), DNA damage response (DDR), genome stability, p53 family members, DNA replication fork progression and translesion synthesis

## Abstract

Maintenance of genome stability is a crucial priority for any organism. To meet this priority, robust signalling networks exist to facilitate error-free DNA replication and repair. These signalling cascades are subject to various regulatory post-translational modifications that range from simple additions of chemical moieties to the conjugation of ubiquitin-like proteins (UBLs). Interferon Stimulated Gene 15 (ISG15) is one such UBL. While classically thought of as a component of antiviral immunity, ISG15 has recently emerged as a regulator of genome stability, with key roles in the DNA damage response (DDR) to modulate p53 signalling and error-free DNA replication. Additional proteomic analyses and cancer-focused studies hint at wider-reaching, uncharacterised functions for ISG15 in genome stability. We review these recent discoveries and highlight future perspectives to increase our understanding of this multifaceted UBL in health and disease.

## 1. Ubiquitin and Ubiquitin-Like Proteins (UBLs)—An Overview

**Figure 1:**
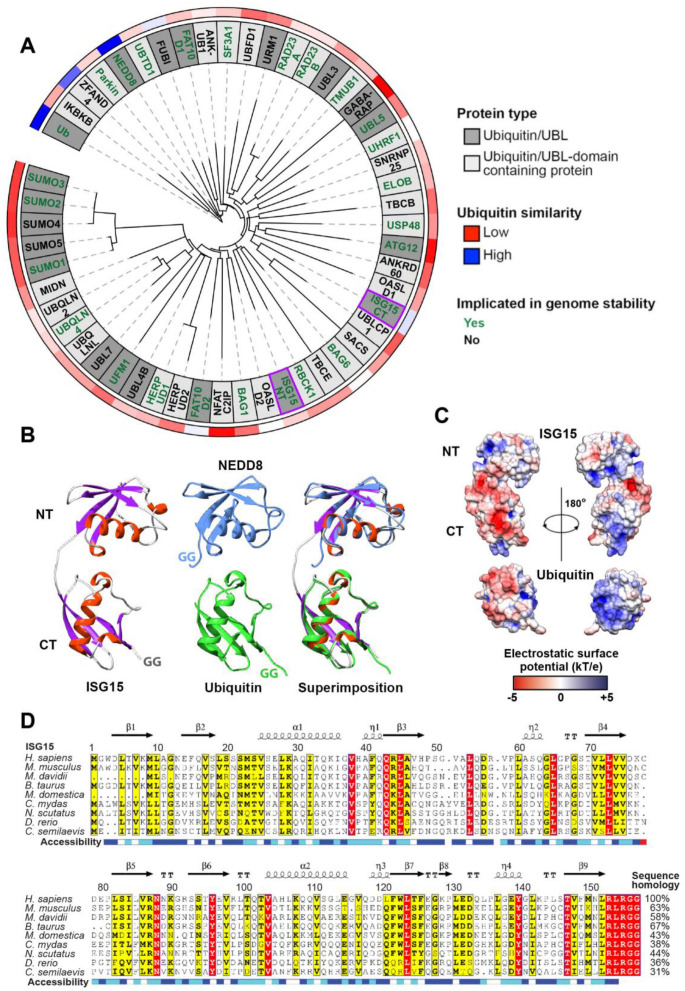
ISG15 comparison with selected ubiquitin/UBLs, ubiquitin-like domains and across species. (**A**) Phylogeny of ubiquitin (Ub), ubiquitin-like proteins (UBLs) and Ub/UBL domains fused to human proteins. Genome stability associations are highlighted in green and apply to the following in addition to ISG15: Ub [3,10], Parkin [47,48,49,50], NEDD8 [11], UBTD1 [51], FAT10 [12], SF3A1 [52], RAD23A and RAD23B [53], TMUB1 (aka HOPS) [54,55,56], UBL5 (aka HUB1) [12], UHRF1 [57,58,59], ELOB (aka TCEB2) [60], USP48 [61,62], ATG12 [63], BAG6 [64], RBCK1 [65], BAG1 [66,67], HERPUD1 [68], UFM1 [12], UBQLN4 [69], SUMO1, SUMO2 and SUMO3 [70]; sequence similarity to ubiquitin is highlighted in the outermost ring ranging from GABARAP (9.59%) in red over a white midpoint to NEDD8 (58%) in blue. Ub/UBL domains are limited to curated UniProt entries. For proteins with multiple Ub/UBL domains, each domain is listed as D1 or D2 with D1 being the closest to the N-terminus except for ISG15. For ISG15, the N-terminus and C-terminus are denoted as NT and CT, respectively (both highlighted in purple boxes). Tree was generated using Phylogeny.fr [71] and visualised with Interactive Tree of Life (iTOL) [72]. (**B**) 3D structures of ISG15 (PDB 1Z2M; lacks C-terminal diglycine), Ub (PDB 1UBQ) and NEDD8 (PDB 1NDD) and their alignment to the N- and C-terminal UBL domains of ISG15. Secondary structures for ISG15 have been highlighted. (**C**) Aligned electrostatic surface potentials of ISG15 and Ub (generated using APBS [73,74,75,76,77,78]). Units are as follows: k (Boltzmann’s constant), T (temperature of calculation: 300 K), e (charge of electron). (**D**) Multiple sequence alignment of the indicated ISG15 orthologues. Bold lettering and yellow boxes represent conserved residues (>70%) considering physicochemical properties of residues whereas red boxes represent residues conserved across all species (100%). Secondary structure (α: alpha helix; η: 3_10_ helix; β: beta-strand; TT: beta-turn) and predicted solvent accessibility (dark blue: accessible; light blue: intermediate; white: buried) of human ISG15 (PDB 1Z2M) is detailed above and below. Percentage of sequence homology to human ISG15 is listed following the sequence. Sequences used are as follows: *Homo sapiens* (AAH09507.1), *Mus musculus* (house mouse; AAB02697.1), *Myotis davidii* (vesper bat; ELK23605.1), *Bos taurus* (cattle; NP_776791), *Monodelphis domestica* (opossum; XP_001372717.2), *Chelonia mydas* (sea turtle; XP_027689314.1), *Notechis scutatus* (tiger snake; XP_026543804), *Danio rerio* (zebrafish; NP_001191098.1) and *Cynoglossus semilaevis* (tongue sole; NP_001287935). Alignments were generated using T-Coffee [79] and visualised with ESPript 3 [80].

Ubiquitylation is one of the most studied post-translational modifications (PTMs) and involves the conjugation of ubiquitin (8.5 kDa), a highly conserved 76 amino-acid protein, primarily onto lysines of target proteins via a three-step ATP-dependent enzymatic cascade formed by an E1-activating enzyme, an E2-conjugating enzyme and an E3 ligase [1]. Ubiquitylation and conjugation with UBLs (UBLylations) are commonly reversible processes catalysed by deubiquitylating enzymes (DUBs) and UBL-specific proteases (ULPs), respectively [1,2,3]. Following the discovery of ubiquitin in 1975, >10 human UBLs have been identified, including several paralogues of small ubiquitin-like modifier (SUMO), neural precursor cell expressed and developmentally down-regulated 8 (NEDD8), interferon-stimulated gene 15 (ISG15, aka G1P2), human leukocyte antigen F locus adjacent transcription 10 (FAT10), ubiquitin-fold modifier 1 (UFM1), ubiquitin-related modifier 1 (URM1), autophagy-related protein 12 (ATG12), autophagy-related protein 8 (ATG8), Finkel-Biskis-Reilly murine sarcoma virus (FBR-MuSV) ubiquitously expressed (FUBI) and ubiquitin-like protein 5 (UBL5) [1,2]. Ubiquitin-like domains also exist as integral parts of proteins with functions often linked to the proteasome (Figure 1A) [4,5]. Importantly, ubiquitylation and UBLylation are key for regulating essential cellular processes including responses to different cellular stimuli, such as genotoxic stress. It is therefore not surprising that deregulation of ubiquitin and UBL systems are linked to a wide variety of human diseases, including cancer and neurodegenerative disorders as well as immune and inflammatory diseases [6,7,8]. As a consequence, components of the ubiquitin/UBL systems represent attractive drug targets for treating these diseases [9]. Although the involvement of ubiquitin, SUMO and NEDD8 in genotoxic stress responses is well established [3,10,11], the roles of the remaining UBLs in this regard are only starting to emerge [12,13]. Indeed, while ISG15 has primarily been associated with antiviral immune responses [14,15], additional non-canonical roles are starting to be uncovered. Herein, we review the emerging roles of the ISG15 system in DNA damage repair/signalling and associated pathways.

## 2. ISG15 and ISGylation

ISG15 was the first UBL to be discovered in 1979, four years after ubiquitin [16,17]. Initially termed ubiquitin cross-reactive protein (UCRP) after its ability to cross-react with ubiquitin antibodies [14], it was later renamed to ISG15 [18,19]. ISG15 bears key features found in all UBLs, namely a β-grasp fold partially wrapped around a short and flexible C-terminal tail terminating in a diglycine by which ISG15 can be conjugated onto substrates (Figure 1B). An aspect of ISG15 distinct from ubiquitin is that, similar to FAT10, it is comprised of two UBL domains [1]. While the N- and C-terminal UBL domains of ISG15 possess only ~30% sequence homology with ubiquitin, they share strikingly similar tertiary structures and display comparable as well as distinct areas of electrostatic surface potentials with ubiquitin (Figure 1C) [8,20,21,22,23]. Interestingly, the two ISG15 UBL domains differ in molecular function, with the solvent-exposed N-terminal domain facilitating ISG15 transfer from E2 to substrate and the C-terminal domain being crucial for E1-mediated ISG15 activation and transthiolation [20,22]. ISG15 orthologues can be found in various organisms although cross-species conservation of amino-acid sequences is relatively low even amongst mammals (Figure 1D) [24,25]. Moreover, the orientation of the two ISG15 UBL domains varies considerably across species [26,27,28], contrasting with the almost 100% cross-species conservation of ubiquitin [29], suggesting that *ISG15* is an evolutionarily diverse and non-essential gene.

**Figure 2:**
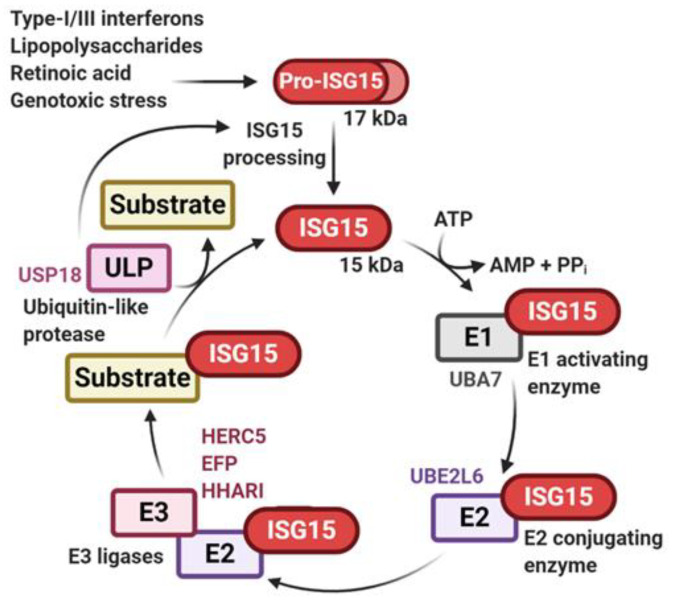
ISG15 conjugation cascade. Expression of ISGylation components is induced by different stimuli as indicated. After processing into its mature form, ISG15 can be conjugated to proteins via a three-step enzymatic cascade comprised of UBA7 (aka UBE1L) as the E1 activating enzyme, UBE2L6 (aka UBCH8) as the E2 conjugating enzyme and either HERC5, EFP (aka TRIM25) or HHARI (aka ARIH1) as the E3 ligase (note that these ligases are diverse in nature encompassing HECT-type, RING-type and HECT-RING hybrid E3 ligases, respectively). The ubiquitin-like protease (ULP) USP18 (aka UBP43) acts as an ISG15-specific protease, cleaving ISG15 from substrates and processing pro-ISG15 into its mature form. Figure created using BioRender.com (2020).

As with ubiquitylation, ISGylation involves a three-step enzymatic cascade (Figure 2). Initially, an inactive 17-kDa precursor of ISG15 is transcribed before being proteolytically cleaved into its 15-kDa mature form [30,31], exposing a highly conserved, C-terminal LRLRGG motif required for canonical conjugation [32]. Initial activation is carried out in an ATP-dependent manner by the ISG15 E1 enzyme UBA7 (aka UBE1L) [33]. ISG15 is then transferred to the active cysteine of the E2 UBE2L6 (aka UBCH8) before being conjugated onto substrates by one of three identified E3 ligases: HERC5 [34], EFP (aka TRIM25) [35] or HHARI (aka ARIH1) [36]. Unlike the ISG15-specific UBA7, UBE2L6 and all three E3s can participate in ubiquitylation events [24,37,38,39,40]. However, while UBE2L6 is capable of ubiquitin E2 activity in vitro [41], a significantly higher affinity for UBA7 over the ubiquitin-specific E1, UBA1 (aka UBE1), suggests that UBE2L6 is ISG15-specific in cells [42]. Removal of ISG15 from substrates and processing of pro-ISG15 [43] can be catalysed by USP18 (aka UBP43) [43,44], which has been confirmed as the major deISGylase in vivo [45,46].

## 3. ISG15: More Than an Antiviral Protein

**Figure 3:**
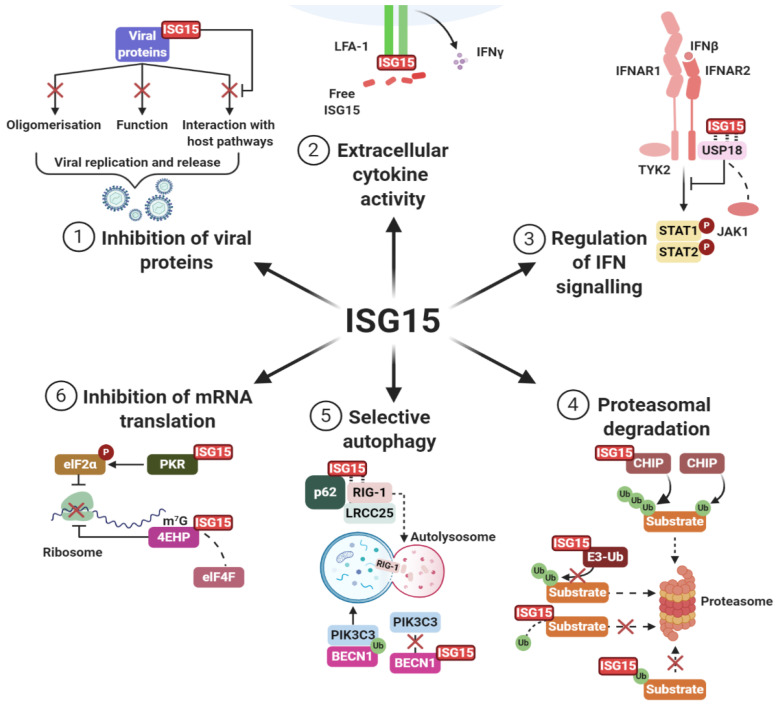
Canonical ISG15 functions in key cellular processes. (1) Inhibition of viral proteins. ISG15 modifies numerous viral proteins and is capable of disrupting their oligomerisation, function and interaction with host pathways. (2) Extracellular cytokine activity. Free ISG15 can be secreted as a cytokine and stimulate IFNγ release through interaction with the LFA-1 receptor. (3) Regulation of IFN signalling. The ISG15-protease USP18 interferes with type-I IFN signalling via direct interaction with the INFAR2 subunit of the IFN receptor. This displaces JAK1 and prevents JAK/STAT signal transduction. Non-conjugated ISG15 binds and stabilises USP18. (4) Inhibition of proteasomal degradation. ISGylation can interfere with ubiquitin-mediated proteasomal degradation through inhibition of E3 ligases, competition for lysine conjugation sites and formation of mixed chains. Alternatively, ISG15 can promote proteasomal degradation through stimulation of CHIP (aka STUB1) activity. (5) Selective autophagy. ISG15 can promote selective autophagy of target proteins. In the case of RIG-1, ISG15 association allows for interaction with LRCC25, facilitating p62-guided RIG-1 degradation via the autophagosome. Conversely, ISGylation of the autophagy-promoting protein BECN1 prevents its activity through disrupting its ubiquitin-mediated interaction with PIK3C3. (6) Inhibition of protein translation. ISGylation of PKR promotes phosphorylation and activation of the translational suppressor elF2α. Similarly, ISGylation of 4EHP increases its affinity to 7-methylguanosine mRNA cap binding, displacing elF4F and inhibiting protein translation. Figure created using BioRender.com (2020).

All components of the ISGylation cascade are transcriptionally induced by type-I/III interferons (IFNs), lipopolysaccharides, retinoic acid, genotoxic stress or other immune activators (e.g., references [18,24,81,82,83,84,85,86,87,88,89,90]). As one of the earliest induced proteins following type-I IFN signalling [18], ISG15 has been investigated extensively regarding its effects on countering viral and bacterial infections. In this regard, ISGylation of viral and host proteins can directly inhibit the functions of viral proteins (Figure 3, Section 1) [15,24]. Additionally, free extracellular ISG15 can act as a cytokine, stimulating IFNγ secretion in natural killer cells [91,92] (Figure 3, Section 2). Moreover, components of the ISGylation cascade such as USP18 can modulate JAK-STAT immune responses in an ISGylation-independent manner (Figure 3, Section 3) [93].

However, ISG15 also displays broader functions in a variety of cellular processes, such as proteasomal degradation. ISGylation was first linked to the proteasome because of a marked increase in ISG15 conjugates upon proteasomal inhibition [94]. ISG15 can inhibit proteasomal degradation by outcompeting ubiquitin for conjugation sites [95,96,97,98], by directly inhibiting ubiquitin E3 ligases [99,100] or by ISGylation of ubiquitin at K29, forming mixed chains, which are ineffective as degradation signals [101]. However, ISG15 can also act in a similar fashion to ubiquitin by targeting certain proteins for proteasomal degradation [102,103,104,105,106]. Furthermore, ISG15 can enhance overall ubiquitylation and substrate degradation [107,108] e.g., by ISGylation of CHIP (aka STUB1), a key E3 ligase in protein quality control [109], which accelerates CHIP activity, thereby increasing activation of the ubiquitin-proteasome system (UPS) (Figure 3, Section 4) [110]. Taken together, these findings closely tie ISG15 to ubiquitin and the proteasome, and suggest complex roles for ISG15 in proteasomal degradation depending on the exact circumstances.

In addition to roles in the UPS, ISG15 has recently emerged as a key modulator of autophagy. For example, several crucial facilitators of selective autophagy interact with free and conjugated ISG15, potentially promoting autophagy of ISG15-conjugates (Figure 3, Section 5) [111]. HyperISGylation can even induce aberrant autophagy under genotoxic stress, at least in certain pathological circumstances [112,113]. By contrast, ISG15 can also have a negative effect on autophagy e.g., by ISGylation, and subsequent inhibition, of positive autophagy regulators (Figure 3, Section 5) [114,115,116]. In certain circumstances ISGylation can also protect proteins from lysosomal degradation, challenging the notion that ISG15 simply serves as a tag for selective autophagy [117]. These studies suggest a sophisticated role for ISG15 in autophagy that varies depending on the cellular context.

In protein synthesis ISG15 can inhibit translation of individual proteins through modification of RNA-binding partners [118], as well as by suppressing global or mRNA-specific translation via ISGylation of key translational regulators (Figure 3, Section 6) [36,119,120]. This activity is largely thought to be an antiviral mechanism whereby translation of newly synthesized viral proteins is restricted via translational suppression following IFN stimulation. In addition, ISG15 may function as a co-translational modifier, targeting nascent viral or misfolded proteins for degradation. Indeed, HERC5 localises to polyribosomes and can broadly target newly synthesized proteins [121], which may also enhance the presentation of antigens on MHC class 1 molecules by bolstering co-translational antigen processing [122]. Further research is required to understand if and how ISG15-mediated inhibition of translation occurs outside of an antiviral context.

ISG15 can also influence numerous other cellular processes, including inhibition of exosome secretion [123], attenuation of hypoxia [124], activation of cytokine secretion [89,91,125,126,127,128] as well as modulation of cytoskeleton dynamics [129,130]. For further insights and recent trends into the more canonical activities of ISG15 we refer the reader to the following reviews [15,131,132]. These findings illustrate the highly diverse impact of ISG15 on a wide variety of cellular processes and highlight its capacity to partake in complex pathway regulation, which also applies to the intertwined processes that make up the DNA damage response (DDR).

## 4. The DNA Damage Response (DDR)

Genome integrity is constantly challenged by intrinsic and extrinsic stressors. Reactive oxygen species (ROS), base pair mismatches—occasionally introduced during DNA replication—and topoisomerase-generated DNA breaks are examples of endogenous sources of DNA damage. Exogenous DNA stressors include ionizing radiation (IR), ultraviolet light (UV) and genotoxic agents such as chemotherapeutic drugs [133,134]. These DNA-damaging agents cause DNA lesions which, if un- or misrepaired, are capable of interfering with essential cellular processes. For instance, high levels of DNA damage in S-phase can halt DNA replication fork progression and cause DNA replication stress [135]. Inaccurate repair of DNA lesions can also give rise to mutations and chromosomal abnormalities which lead to genomic instability, a hallmark of cancer [136]. For the survival and normal functioning of a mammalian organism, maintaining genome stability is therefore critical for accurate transmission of genetic information and to prevent prevalent diseases. In fact, carriers of hereditary DNA repair deficiencies can be predisposed to tumourigenesis, immunodeficiency, neurodegeneration, infertility and premature ageing, highlighting the broad importance of genome integrity to human health [133,134,136].

**Figure 4:**
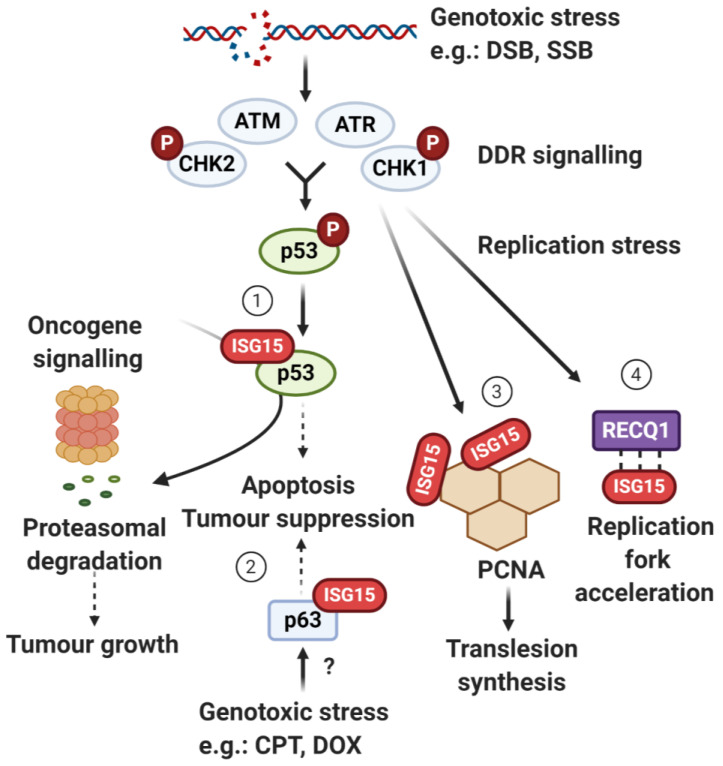
Key aspects of the DNA damage response (DDR) and associated pathways regulated by ISG15. Following exposure to genotoxic stress, DDR signalling is initiated via the activation of apical DDR kinases including ATM and ATR and their targets CHK2 and CHK1, respectively, which in turn activate downstream pathways, such as p53-mediated apoptosis and tumour suppression, or processes that facilitate DNA replication past lesions including translesion DNA synthesis and regulation of replication fork progression. ISG15 intersects with these pathways at multiple points: (1) DNA damage-induced activation of p53 induces the expression of the ISG15 system. ISGylation of p53 in multiple positions can target p53 for proteasomal degradation to promote tumour growth, or, alternatively, mediate cell-cycle arrest, apoptotic responses and ultimately tumour suppression depending on the exact circumstances. ISGylation of p53 has also been linked to oncogene-mediated transformation by targeting p53 for degradation via the 20S proteasome. (2) In order to prevent tumourigenesis, genotoxic stress caused by chemotherapeutic drugs can stimulate ISGylation of p63 isoforms independently of p53 through yet not fully understood mechanisms. In addition, ISGylation of PCNA (3) and non-covalent interaction of ISG15 with RECQ1 (4) function in translesion DNA synthesis and replication fork acceleration, respectively, in cells undergoing replicative stress. CPT: camptothecin; DOX: doxorubicin; DSBs: DNA double-strand breaks; SSBs: DNA single-strand breaks; P: phosphorylation. Figure created using BioRender.com (2020).

To maintain genome stability, cells have evolved the DDR, a collection of pathways which coordinates the detection, signalling and repair of DNA lesions, and arrests cell-cycle progression to allow time for repair [133,134]. DDR sensors activate the DDR signalling network, which is orchestrated by phosphoinositide 3-kinase-like kinases (PIKKs) including the master regulator kinases ataxia telangiectasia mutated (ATM) and ataxia telangiectasia and Rad3 related (ATR; Figure 4). PIKKs amplify DDR signalling by phosphorylating and activating other DDR factors, such as the checkpoint kinases CHK1 and CHK2 as well as the downstream tumour suppressor p53 which regulates the transcription of hundreds of DDR effectors to induce G1 cell-cycle arrest, senescence and/or apoptosis depending on the type of DNA damage and cellular context [137,138].

The DDR also coordinates DNA replication and repair with cell-cycle progression to preserve genome stability. S-phase is particularly vulnerable to detrimental effects of bulky DNA lesions, which cause replication fork stalling. For instance, the S-phase checkpoint elicits protective ATR/CHK1-mediated responses including the repression of unfired origins of replication, the stabilisation of replication forks and the inhibition of entry into mitosis if DNA is not fully replicated, thus constituting the surveillance mechanism that prevents genome instability upon replication stress. The attenuation of S-phase checkpoint signalling and recovery from DDR-induced cell-cycle delay is critically dependent on post-replication repair mechanisms to resolve stalled replication forks and allow for S-phase progression on damaged genomic DNA templates [139]. Translesion DNA synthesis (TLS), a DNA damage tolerance mechanism, allows cells to bypass DNA lesions that would otherwise block the replication machinery while tolerating their repair at a later stage, thus avoiding the collapse of replication forks. One central TLS component is the sliding clamp proliferating cell nuclear antigen (PCNA), a replication processivity factor that supports the assembly of DNA replication and repair proteins [140]. Among other replicating factors, the RECQ1 helicase is critically involved in replication fork restart under replication stress conditions, cell-cycle progression and genotoxic stress resistance [141,142]. Importantly, PTMs are crucial for coordinating the above processes and therefore are key to a functioning DDR [3,10,133,143]. In addition to PTMs such as ubiquitin and the UBLs SUMO and NEDD8, ISG15 has emerged as an important regulator of genome stability through covalent modification of—or non-covalent interaction with—target proteins involved in key aspects of the processes described above, ranging from p53 and p63 signalling to TLS and replication fork progression (Figure 4, sections 1–4) [23,85,86,88,104,144].

## 5. ISG15 System and p53—A Complex Relationship

p53 coordinates cellular responses to stressors, such as DNA damage, telomere erosion, oncogene activation and hypoxia [138]. Under normal conditions, p53 levels are kept low primarily by targeting p53 for proteasomal degradation via its interaction with, and ubiquitylation by, the ubiquitin E3 ligase Mdm2 [145,146,147]. Under stress conditions, the interaction of p53 with Mdm2 is disrupted by phosphorylation and acetylation leading to p53 stabilisation and activation. Activated p53 then binds to p53-responsive elements (*p53REs*) for transcriptional activation of target genes (e.g., *BAX*, *p21* and *PUMA*) that modulate cell-cycle arrest, senescence and/or apoptosis [148,149,150]. ISG15 expression has been linked in different ways to p53-mediated cellular responses [12,88,104,107,144,151,152,153].

### 5.1. ISG15 as a p53 Degradation Signal

**Figure 5:**
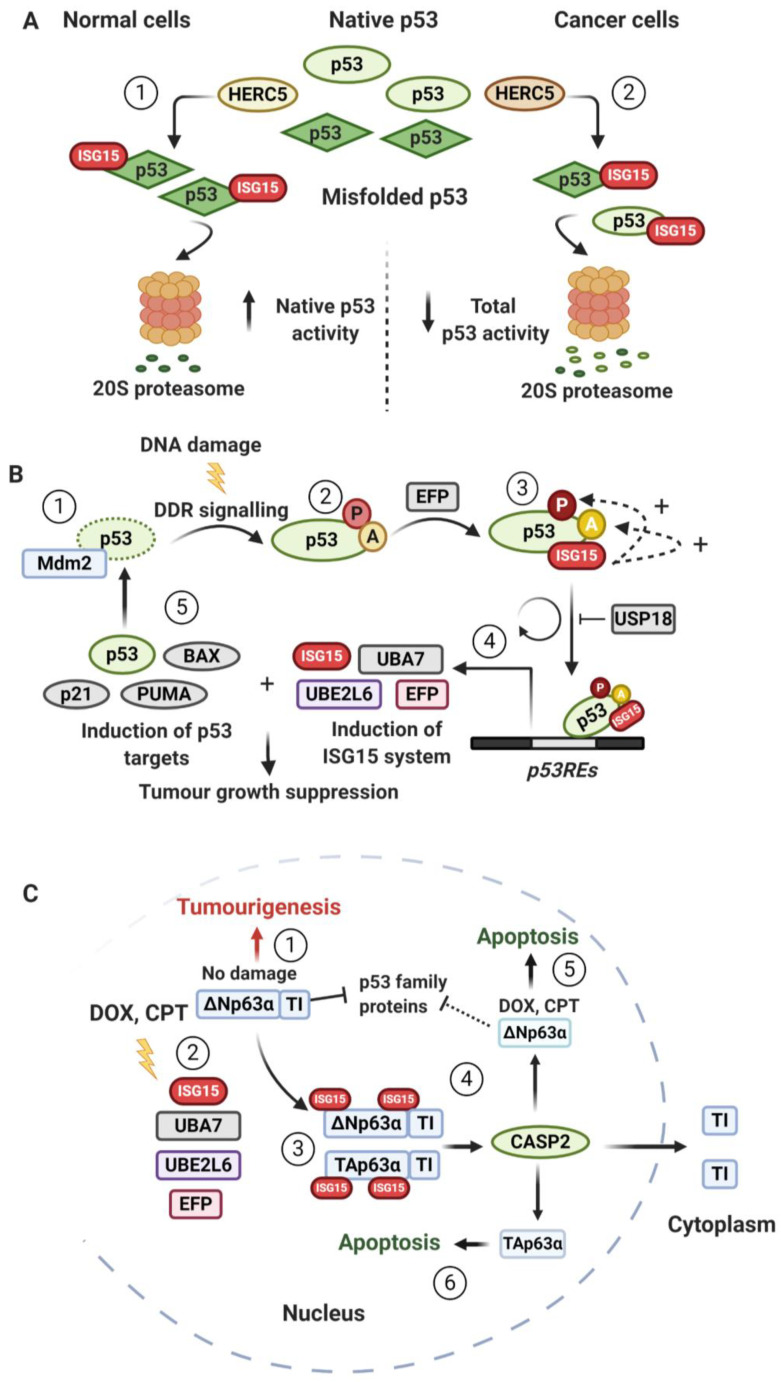
Pleiotropic functions of p53 ISGylation. (**A**) HERC5-mediated p53 ISGylation. (1) In non-transformed cells, HERC5 (light brown) mediates ISGylation of p53 to primarily remove dominant-negative/misfolded p53 via the 20S proteasome, thereby increasing native p53 activity. (2) In cancer cells, oncogene proteins, such as *SRC*, *RAS* and *MYC*, enhance the interaction between HERC5 (dark brown) and ISG15, leading to indiscriminate modification of native and misfolded p53 and an overall reduction of total anti-tumoural p53 activity. (**B**) EFP-mediated p53 ISGylation. (1) p53 is associated with Mdm2 under normal cellular conditions. (2) Upon DNA damage, p53 is phosphorylated (orange, P) and acetylated (yellow, Ac) via DNA damage response signalling pathways, resulting in dissociation of p53 from Mdm2 and subsequent p53 stabilisation. (3) EFP conjugates ISG15 to stabilised p53 at lysines K291 and K292, which increases the phosphorylation and acetylation status of p53 (denoted as +), as well as its ability to bind p53 responsive elements (*p*53*RE*s). (4) This induces the expression of p53 targets, such as *p21*, *BAX*, *PUMA*, *p53* itself and ISGylation factors. (5) In a positive feedback loop, increased expression of ISGylation factors accelerates p53 ISGylation and transactivation, leading to tumour growth suppression. This loop is deactivated by USP18 (aka UBP43) via deISGylation of p53, leading to p53 destabilisation. Note that levels of Mdm2 are also increased with p53 transactivation, which further contribute to downregulating the cycle. (**C**) Abrogation of the oncogenic functions of ∆Np63α, an isoform of p53 family member p63, by ISGylation. (1) The tumourigenic protein ∆Np63α is overexpressed in human epithelial cancers and in the absence of external DNA-damaging agents, acts by blocking the transactivation (TA) of p53 family members, such as TAp63α and TAp63γ, with its transactivation inhibitor domain (TI). (2) DNA damage caused by doxorubicin (DOX) or camptothecin (CPT) induces the ISG15 and its conjugation system, and (3) the ISGylation of ∆Np63α at lysines K139 and K324. Under the same conditions, TAp63α is also ISGylated at lysines K194 and K397. (4) These events lead to CASP2-mediated cleavage of ∆Np63α at aspartates D452, D469 and D489 and of TAp63α at aspartates D507, D524 and D544, as well as subsequent cytoplasmic release of their TI domains. (5) Cleaved ∆Np63α can no longer inhibit the transcriptional activities of other p53 family members, such as TAp63α and TAp63γ, thus facilitating their anti-tumourigenic functions e.g. via mediating apoptosis. (6) The N-terminal fragment of TAp63α containing the TA domain, but deprived of the TI domain, is relieved from self-suppression leading to anti-tumourigenic effects e.g., via the expression of its downstream apoptotic genes (6). CASP2: caspase 2. Figure created using BioRender.com (2020).

HERC5-mediated ISGylation of p53 has been reported as a proteasomal degradation signal (Figure 5A) [104]. In non-transformed cells, because of the low expression of the ISGylation system, the conjugation of ISG15 to p53 is less prominent and primarily targets misfolded/dominant-negative p53 (Figure 5A, Section 1, left). Indeed, ISG15 deletion results in the accumulation of misfolded/dominant-negative p53 and suppression of overall p53 activity, leading to decreased DNA damage-induced senescence, accelerated cell proliferation and lowered p21 expression. *ISG15^−/−^* mice similarly showed increased cell proliferation in vivo, while mice-derived cells displayed reduced p53 target expression and downregulated apoptosis. Likewise, ISG15 deficiency leads to the accumulation of misfolded dominant-negative p53 in the context of HIV infection [153].

In transformed cells, ISG15 overexpression has been detected in several cancer settings and has been linked to tumourigenesis [95,100,117,129,154,155,156,157,158,159,160,161,162,163,164,165]. p53 ISGylation can be upregulated by oncogenes, such as *SRC*, *RAS* and *MYC*, e.g., via SRC-mediated phosphorylation of p53 positively regulating HERC5 binding to, and ISGylation of, cytoplasmic p53. In transformed cells, both native and misfolded p53 are targeted for degradation by ISG15, reducing the overall p53 levels (Figure 5A, Section 2, right) [144].

As a result, ISG15 deficiency can upregulate p53 transactivity and thus reduce cellular proliferation/tumourigenic potential of transformed cells and lung tumour formation/growth in a *KRAS* cancer mouse model [144]. ISG15 deficiency can also enhance DNA damage-induced transcriptional activation of p53 in cancer cells exposed to different genotoxic agents. In line with the potential cross-talk between ubiquitin and ISG15 and a direct role for ISG15 in p53 degradation, simultaneous suppression of p53 ubiquitylation and ISGylation can potentiate the inhibitory effects on cell proliferation. Therefore, oncogene-mediated transformation of cells may lead to ISG15-dependent degradation of p53 and tumourigenesis, highlighting the distinct effects of ISG15-modulated p53 degradation depending on cellular context [144].

### 5.2. p53-Mediated Induction of ISG15 System

ISGylation does not only impact p53 levels and function, but p53 itself can regulate the ISG15 system. ISGylation is upregulated by genotoxic stressors, such as UV irradiation, doxorubicin (DOX) and camptothecin (CPT) because of the presence of *p53REs* in the *ISG15*, *UBA7*, *UBE2L6* and *EFP* promoters which are induced by p53 independently of IFN-I signalling (Figure 5B) [83,84,85,86,88]. Inhibition of ATM/ATR in several *p53^+/+^* but not *p53^−/−^* cancer cell lines therefore abrogates the induction of ISGylation components, consistent with genotoxin-induced ISGylation of p53 being promoted by PIKKs via their known role of p53 activation [88,137]. However, ATM has also been suggested to suppress the ISG15 system [97], pointing towards nuanced layers of control depending on the exact circumstances. It is noteworthy that HERC5 lacks a *p53RE* in its promoter and is not induced by genotoxic agents. Since HERC5 is physically associated with polyribosomes and modifies a wide range of newly synthesized proteins in a co-translational manner, HERC5 may ISGylate newly synthesized/unstructured p53 in a non-specific manner [88,121].

### 5.3. ISG15 Enhances p53 Transactivity

DNA damage-activated p53 can be ISGylated at K291/K292 by EFP (Figure 5B). These ISGylations promote p53 transactivity by enhancing p53 phosphorylation and acetylation and consequently, the affinity of p53 to the *p53REs* of its own gene and downstream targets (e.g., *p21*, *BAX*, *MDM2*), ultimately modulating cell-cycle arrest and apoptosis. In a positive feedback loop, the ISG15 conjugation system is also upregulated by p53 ISGylation to further potentiate p53 transactivity and downregulated by USP18-mediated deISGylation of p53. In cellular assays EFP-mediated p53 ISGylation increased p53 stability, while ISG15 or EFP depletion, or expression of K-to-R p53 mutants significantly decreased the DNA damage-induced p53 responses. Furthermore, p53 ISGylation inhibited cellular proliferation and tumour growth in mice via p53 tumour suppressive functions, therefore supporting a role for ISG15 in preventing tumourigenesis under genotoxic stress [88].

Taken together, these findings reveal a pleiotropic and complex relationship between ISG15 and p53 that takes place in the presence of a gamut of other PTMs likely to compete for target residues. It appears that the cellular responses and precise ISGylation sites on p53, as well as the contributing ISGylation components, depend on various factors including cellular context and extracellular stimuli. What is clear from the above is that ISGylation plays important and diverse roles in helping cells to fine-tune and adapt downstream p53-mediated DDR processes to maintain genome stability.

## 6. ISGylation of ∆Np63 and Tumourigenesis

The p53 protein family comprises the p53, p63 and p73 transcription factors [166]. Alternative promoter usage generates two p63 transcripts, one including an N-terminal transactivation domain (TA) and the other lacking the TA domain (ΔN). Alternative splicing of TAp63 and ΔNp63 generates unique p63 C-termini, namely α, β, γ, δ and ε. Similar to p53, TAp63 isotypes function as tumour suppressors by inducing cell-cycle arrest and apoptosis via p53-responsive genes. The p63α isotypes also contain a C-terminal transactivation-inhibitory (TI) domain, which can suppress the transactivity of p53 family members by binding to their TA domains [166,167]. In cancer cells with elevated ΔNp63α levels, such as human epithelial cancers, the tumour-suppressive functions of the TA isotypes are inhibited by the dominant-negative action of ΔNp63α (Figure 5C, step 1). This suppression makes cells resistant to apoptosis, causing uncontrolled cell proliferation and tumour formation [166,167].

Upon treatment with DOX and CPT, ΔNp63α is ISGylated at K139 and K324 in various cell lines (Figure 5C, steps 2–3) [85]. Through an unknown mechanism, caspase 2 (CASP2) is then activated to specifically recognise ISGylated ΔNp63α and cleave off its TI domain. The N-terminal ΔNp63α fragment lacking the TI domain subsequently fails to suppress transactivation of the other TA isotypes. The C-terminal ΔNp63α fragment, although retaining the TI domain, is exported to the cytoplasm, abolishing its dominant-negative function towards nuclear p53 family members (e.g., TAp63γ; Figure 5C, steps 4–5, top). In cellular assays, ΔNp63α ISGylation abrogates its ability to inhibit both apoptosis and RAS-driven senescence as well as to induce cell growth and tumour formation. Conversely, ISG15 depletion, K-to-R mutations of ΔNp63α ISGylation sites, or D-to-A mutations of CASP2 cleavage sites markedly potentiate ΔNp63α-mediated anchorage-independent cell growth and tumour development in vivo [85].

TAp63α is also ISGylated at K194 and K397 and cleaved by CASP2, followed by export of its C-terminal TI domain to the cytoplasm. The N-terminal fragment of TAp63α, containing the TA domain but lacking the TI domain, becomes unsuppressed and thereby capable of inducing expression of its downstream apoptotic genes (Figure 5C, steps 2–4,6, bottom). According to this model, ISG15 conjugation to ΔNp63α and TAp63α plays a critical role in maintaining genome stability and suppressing tumourigenesis, particularly in epithelial cancer cells under genotoxic stress. The model also provides a molecular mechanism for the use of chemotherapeutic drugs to treat ΔNp63α-mediated cancers [85].

Intriguingly, DNA damage-induced ISGylation of ΔNp63α can occur in cells expressing mutated non-functional p53. Therefore, p53-independent induction of the ISG15 system in these cells is likely under the control of other signalling pathways, such as those mediated by Notch [85,88]. Alternatively, a second TA domain present in ΔNp63α lacking the N-terminal TA domain could potentially regulate the expression of a distinct subset of genes [167]. Therefore, ΔNp63α itself might induce the ISG15 system to mediate its own ISGylation [88].

## 7. Translesion DNA Synthesis (TLS)—A New Terminator Model

In order to maintain faithful transmission of genetic information, cells need to replicate their genome accurately and facilitate efficient and faithful repair of DNA damage, such as nucleotide mismatches occasionally introduced during DNA replication [168]. PCNA is a critical processivity factor and a scaffold for recruiting the replication machinery. Moreover, PCNA is important for DNA lesion bypass in the TLS pathway by serving as a platform for recruiting DNA damage tolerance factors, making PCNA a key regulator of genome stability [140].

When replicating cells encounter DNA damage, PCNA undergoes multiple PTMs including RAD6/RAD18-mediated monoubiquitylation at K164 to initiate TLS by exchanging replicative polymerases, such as Pol δ, with DNA damage-tolerant polymerases, such as Pol η [140,169,170,171]. TLS polymerases then bypass damaged DNA, allowing replication fork progression to occur without immediate damage removal and risk of fork collapse [87,140,171]. However, TLS polymerases lack proofreading activity, can introduce nucleotide mismatches and, as a result, are potentially mutagenic. Consequently, error-prone TLS polymerases need to be promptly released from PCNA after DNA lesion bypass to prevent excessive mutagenesis [171].

**Figure 6:**
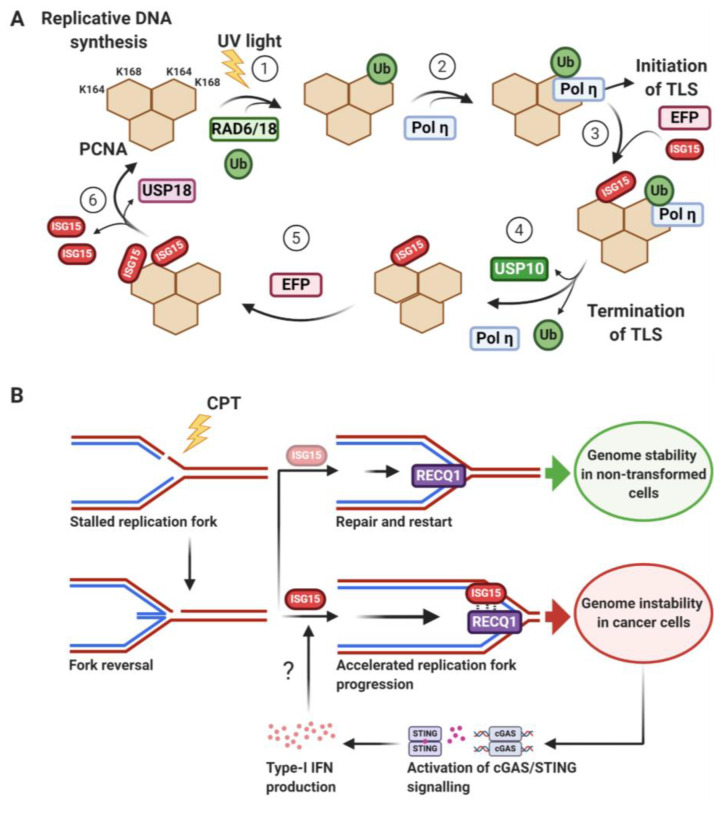
ISG15 in DNA synthesis—replicating past lesions. (**A**) Termination of translesion DNA synthesis (TLS) by ISGylation of PCNA. Under non-stressed conditions, PCNA serves as a processivity factor for replicative DNA synthesis. In response to certain types of DNA damage (e.g., UV light) PCNA is key to initiating and terminating TLS as follows: (1) PCNA is monoubiquitylated by the RAD6/RAD18 E3 ligase complex at K164 in one of its three identical subunits, which (2) recruits the translesion polymerase Pol η to carry out TLS. (3) After bypass of the lesion, EFP ISGylates PCNA at lysine K168. (4) ISGylation of PCNA triggers recruitment of the deubiquitylase USP10, which in turn deubiquitylates PCNA and releases Pol η to avoid UV-induced mutagenesis. (5) A further ISGylation step of PCNA by EFP at lysine K164 likely prevents additional cycles of monoubiquitylation. (6) Finally, USP18 (aka UBP43) deISGylates PCNA and allows reloading of replicative DNA polymerases as well as resumption of replicative cell replication. (**B**) ISG15 accelerates replication fork progression. Association of ISG15 with the DNA helicase RECQ1 accelerates replication and promotes stalled replication fork restart. While multiple mechanisms of replication fork restart and reversal exist, single-stranded DNA (ssDNA) is typically exposed to and bound by RPA, which triggers events leading to DNA loading of PCNA and its polyubiquitylation by UBE2N (aka UBC13). Upon fork reversal complementary nascent DNA strands can be used as a template to bypass DNA lesions, such as those caused by camptothecin (CPT). Reversed forks are stabilised by different factors before being restarted by RECQ1 although the exact mechanisms behind this are unclear [172]. ISG15 accelerates RECQ1 activity leading to increased rates of DNA synthesis, potentially interfering with coordination of fork restart, thereby promoting genome instability. In cancer cells overexpressing ISG15, this increased genome instability has the potential to create a positive feedback loop mediated by cGAS/STING signalling whereby type-I interferons are upregulated following DNA damage, although the exact underlying mechanisms remain to be determined e.g. in light of recently described effects of cGAS/STING on replication fork progression [173]. Ub: Ubiquitin. Figure created using BioRender.com (2020).

ISGylation of PCNA plays a key role in TLS termination (Figure 6A) [86]. Upon DNA damage by e.g., UV light, PCNA is first monoubiquitylated at K164 in one of its three identical subunits to promote recruitment of Pol η and initiate TLS (Figure 6A, steps 1–2) [171]. After DNA lesion bypass, EFP ISGylates a different subunit of monoubiquitylated PCNA at K168 (Figure 6A, step 3).

ISGylated and ubiquitylated PCNA is subsequently deubiquitylated by USP10 which, in turn, triggers the release of Pol η from PCNA for TLS termination (Figure 6A, step 4). EFP is able to conjugate an additional ISG15 to PCNA at K164, thus forming diISGylated PCNA with two ISG15 molecules in the same subunit, likely for preventing additional cycles of PCNA monoubiquitylation and subsequent recruitment of Pol η (Figure 6A, step 5) [86,87]. The exact order of some of these events and their duration of functioning are not entirely clear. Moreover, it will be interesting to see how ISGylation of PCNA integrates with potential additional/alternative pathways to help terminate TLS [174,175]. Eventually, ISG15 expression is downregulated and PCNA is deISGylated by USP18 for reloading of replicative DNA polymerases and resumption of DNA replication (Figure 6A, step 6). Therefore, the sequential modification of PCNA (monoubiquitylation, ISGylation, deubiquitylation, and deISGylation) occurs in a timely manner after UV irradiation. ISGylation-defective K-to-R PCNA mutants or depletion of any of ISG15, EFP, or USP10 leads to persistent recruitment of monoubiquitylated PCNA and Pol η to UV-induced DNA damage sites, causing an increase in UV-mediated mutation frequency and reducing PCNA interaction with the Pol δ catalytic subunit in vitro [86]. These findings establish a crucial role for PCNA ISGylation in termination of error-prone TLS after DNA lesion-bypass and in preventing excessive mutagenesis to maintain genome stability.

## 8. ISG15 in Replication Fork Progression

Error-free DNA replication is a central pillar in the preservation of genomic integrity. As new DNA is synthesised, cells must carefully balance speed and accuracy of replication against the distribution and availability of key replication factors. Any disruptions in this delicate process can lead to replication stress, often presenting as slowed or stalled replication forks [176].

Recently, a role for ISG15 in replication processivity has been revealed (Figure 6B) [23,177]. Increased levels of ISG15 resulted in, or were representative of, accelerated fork progression and reduced sensitivity to CPT-induced fork slowing. The phenotype was largely independent of ISGylation, primarily relying on non-covalent interaction between ISG15 and the DNA helicase RECQ1 [23].

This study complements the role of ISG15 in translesion synthesis, with both mechanisms acting as a means of replicating past genomic stress. Moreover, it offers an additional perspective regarding the effects ISG15 can have on genome stability. RECQ1 is a key helicase responsible for restarting stalled replication forks, a process that if underregulated promotes DSB formation [142]. ISG15 seemingly encourages this activity as its increased expression, when uncoupled from the induction of its conjugation system, can induce DSBs and chromosomal aberrations as a likely result of replication fork acceleration. Interestingly, similar effects have previously been observed following the inhibition of poly(ADP-ribose) polymerase (PARP) [178], a key negative regulator of RECQ1 and common drug-target in the treatment of HR-deficient cancers. Given that ISG15 overexpression and PARP inhibition were reported to have no additive effect on fork acceleration, it is possible that the two may operate through similar mechanisms. Additionally, elevated ISG15 sensitised cancer cells to both CPT and the PARP inhibitor olaparib, raising intriguing possibilities regarding the treatment and stratification of cancer patients.

The capacity for highly expressed ISG15 to promote genomic instability through RECQ1 interaction contrasts with the protective function of ISG15 as a facilitator of DNA damage tolerance through covalent modification of PCNA [86]. A possible explanation for these different effects could be that differential ISG15 functions are dependent on the amount of free ISG15 in the cell available to impact on RECQ1. The level of free ISG15 is likely influenced by the amount and functionality of ISG15 conjugating/deconjugating factors versus the level of ISG15 itself, which can both be impacted by the type and quantity of DNA-damaging agents, as well as cell type-specific adaptations. Moreover, the type of genomic stress caused may also be a contributing factor per se, with the potential for ISG15-mediated fork restart/acceleration by RECQ1 and TLS termination being directed by the specific nature of the lesion. Taken together, these findings highlight a nuanced role for how covalent and non-covalent ISG15 interactions with DDR factors define the responses to genotoxic stress in distinct ways under different conditions. These mechanisms may further depend on various other PTMs, protein–protein interaction crosstalk and/or IFN responsiveness. It will be interesting to see how these roles can be put in context with other genome stability pathways likely regulated by ISG15, as outlined below.

## 9. Further Roles in Genome Stability—Bright Prospects for ISG15

Hints of further roles for ISG15 in various genome stability pathways including the DDR, cell-cycle regulation and telomere-associated processes are emerging. For example, differing ISG15 conjugates after DNA-damaging agents and IFN induction suggest uncharacterised ISG15 roles that are independent of an innate immune response [88]. Moreover, ISG15 expression is often perturbed in cancer cell lines and can impact on drug resistance [179]. Indeed, in concordance with its role in replication fork progression, ISG15 is a key determinant of sensitivity to topoisomerase poisons in certain breast, lung and gastric cancer contexts, with higher ISG15 expression being associated with increased sensitivity [180,181,182,183]. By contrast, overexpression of ISG15 can confer gemcitabine resistance in pancreatic cancer cells [161]. Additional studies investigating IFN-driven drug resistance signatures also identify ISG15 as a key marker for resistance to genotoxic therapies involving chemotherapeutic drugs, such as DNA methyltransferase (DNMT) or histone deacetylase (HDAC) inhibitors, or radiotherapy [118,152,184,185,186,187,188]. While the roles of ISG15 in drug sensitivity are unclear, these studies not only hint at functions for ISG15 in genome stability, but also touch on the possibility of interplay between the innate immune system and the DDR.

While telomeres predominantly act as protective chromosome ends to maintain genome stability, they are also capable of regulating expression of specific genes e.g., through long-range intrachromosomal loop structures. This phenomenon is termed telomere position effect over long distances (TPE-OLD) [189]. ISG15 has emerged as a gene regulated by TPE-OLD, with increased ISG15 expression being observed following telomere shortening independent of DDR or IFN signalling [189,190]. Additional studies have supported this, reporting an inverse correlation between ISG15 expression and telomere length [191,192,193]. The biological purpose of this regulatory mechanism is not well understood, but it is possible that TPE-OLD-regulated genes act as a means of monitoring telomere length before the need to initiate the DDR. If this is the case, ISG15 could potentially have roles in telomere maintenance, adding another layer of how ISG15 may contribute to genome stability.

Multiple studies have suggested the involvement of ISG15 in cell-cycle regulation. For example, ISGylation of cyclin D1, the primary cyclin involved in G1-phase progression, promotes its degradation in lung cancer cell lines [103]. ISGylation can also promote degradation of FOXO3a, another key cell-cycle regulator [106], and UBA7 deficiency in murine haematopoietic progenitor cells increases G2/M-phase blockage following transplantation, suggesting a role in stress-induced cell-cycle regulation [194]. Moreover, basal levels of ISG15 and USP18 can dynamically regulate the activity of SKP2, a key ubiquitin E3 involved in S-phase progression [195], and deregulation of ISG15 or USP18 consequently can lead to considerable changes in cell-cycle distribution [107,196].

Proteomic studies investigating ISG15 interactors have identified a considerable number of potential targets relevant to genome stability, the majority of which remain uncharacterised (Table 1) [23,38,85,86,197,198,199]. DDR-relevant ISG15 substrates include, for example, the helicase XPD (aka ERCC2) [199], DDB2 [197] and more recently DDB1 [23] with crucial DDR roles in nucleotide excision repair (NER) [200]. As the ISG15 conjugation system is induced by UV damage [86], it will be interesting to investigate the potential involvement of ISG15 in NER processes. Another validated ISG15 target is PML-RARα, an oncogenic fusion protein and hallmark of acute proteolytic leukaemia (APL) [102,201]. Given that PML nuclear bodies can act as ATM/ATR-regulated DNA damage sensors [202] and that PML colocalises with the DDR factor TOPBP1 at single-strand breaks (SSBs) [203], ISGylation of PML could have unidentified impacts on the DDR and genome stability. Overall, these findings suggest bright prospects for ISG15 in a variety of DDR pathways that remain to be uncovered.

## 10. Conclusions

Given the emerging roles of ISG15 in genome stability, exciting opportunities for new areas of study have arisen. For example, the crosstalk between ISG15 and ubiquitin in genome maintenance represents an interesting avenue for future investigation. Indeed, ISG15 can directly bind and regulate ubiquitylating enzymes [100,252] and various E2s and E3s relevant to genome stability, such as UBE2N [213,253] and CBX4 [221,254], have come up as putative or validated ISG15 targets (Table 1). In fact, ISGylation of UBE2N can inhibit its catalytic ubiquitylation activity [255], and non-canonical conjugation of ISG15 to UBE2N via disulphide bridging has also been observed [256]. While these effects could hamper DNA repair, the existence of complex UBL crosstalk may prevent this from happening. Future studies are required to investigate the potential interplay between ISG15 and other UBL cascades in the DDR and associated pathways. In this regard, emerging technologies capable of performing proteomic studies with improved spatiotemporal resolution will prove beneficial. The advent of new sensitive platforms, such as inducible proximity labelling, photo cross-linking, E2-thioester-driven and related approaches, to identify the weak and transient interactions characteristic of the ubiquitin/UBL systems and the DDR hold considerable promise here [91,257,258,259,260].

Perhaps the most prevalently discussed aspect of ISG15 is its inextricable link to type I IFN signalling. While IFN-independent roles are emerging, to what degree these roles are annexed from the most biologically relevant functions of ISG15 remains to be determined. In recent years, various links between the innate immune system and the DDR have been reported. cGAS-STING signalling in particular has garnered attention, stimulating the innate immune system in response to both viral infection and DNA damage [261]. This is consistent with crucial DDR proteins displaying dual functions in innate immunity [262], and vice versa, with type-I IFN signalling promoting the initiation of the DDR in certain cellular contexts [263]. Future research will shed light on if and how exactly ISGylation contributes to the interplay of these two systems.

The hugely diverse network of PTMs, critical to the DDR and other genome stability pathways, makes defining the downstream readers that non-covalently bind to and action the PTMs into precise cellular activities a high priority for increasing our knowledge of how genome integrity is maintained in the future. In addition, non-covalent interactions with free ISG15 can regulate important pathways, such as replication fork progression [23]. Despite this importance, only a few non-covalent ISG15-binding proteins have been revealed with the most recent, RECQ1, being intricately linked to the DDR [23,91,111,195,252,264,265]. Given the indications of distinct molecular functions of the two UBL domains of ISG15 [20,22,132], it will be exciting to see if such specific functions are more widely mediated by ISG15 interactors binding preferentially to one and/or the other UBL domain. Moreover, the identification of mixed ubiquitin/ISG15 chains [101] and the possibility of poly-chain ISGylations (e.g., references [85,86,88,108,266]) in one or multiple positions on target proteins represent additional opportunities for sophisticated signal integration and recognition via topology-specific ISG15-binding proteins. Further investigation into non-covalent interactors of ISG15 topologies will provide mechanistic insights into the multifaceted functions this UBL plays in a wide variety of cellular pathways. Taken together, it is clear that ISG15 is far more than a simple immunological analogue of ubiquitin. Indeed, understanding the functions of ISG15 in response to genotoxic stress is fundamental for enhancing our knowledge of how genome integrity is maintained, and may thus help prevent, or better treat, the various diseases associated with ubiquitin and UBL defects in the future.

## Figures and Tables

**Table 1:** Selected validated/candidate ISG15 interactors relevant to DNA damage response and beyond.

Experimentally Validated Targets/Interactors	
Targets/Interactors (Targeted Residues)	Roles in DDR/Associated Pathways
CHIP (aka STUB1) (K143) [110]	Regulates proteins involved in BER [204] and cell cycle arrest [205]
Cyclin D1 [103]	Gatekeeping cyclin for DNA replication/ roles in HR [206]
p53 (K291, K292 among others) [88,104]	Master regulator of DNA damage response [207]
p63: ΔNp63α (K139, K324) [85]; TAp63α (K194, K397) [85]	Genome stability/instability, particularly in epithelial cancer cells under genotoxic stress, and depending on isotype [85]
Parkin (K349, K369) [108]	Promotes NER [47] and has important roles in mitosis [49]
PCNA (K164, K168) [86]	Facilitates TLS and has roles in mismatch repair [208]
PML-RARα [102]	Disrupts of PML nuclear body formation important for HR [209]
PTEN [210]	Roles in DSB repair and NER [211]
RECQ1 [23]	DNA helicase in replication stress response [212]
UBE2N (K92) [199]	Roles in DSB repair, NER and TLS [213]
Ubiquitin (K29) [101]	Roles in various DNA repair and signalling pathways [3,10,214]
VCP [198]	DSB repair through extraction of ubiquitylated substrates [215]
XPD (aka ERCC2) [199]	Helicase for NER [216]
**Candidate Targets/Interactors**	
**Targets/Interactors**	**Roles in DDR/Associated Pathways**
AHNAK [197]	Interacts with NHEJ proteins and may facilitate strand ligation [217]
ARID5B [197]	Involved in chromatin organisation and recruited to DNA damage sites [218]
ATXN2 [197]	Suggested protection against oxidative stress/potentially harmful R-loops [219]
CBX1 (aka HP1γ) [23]	Likely promotes recruitment of repair factors in various pathways
CBX3 (aka HP1β) [23]	Likely promotes recruitment of repair factors in various pathways [220]
CBX4 [197]	Mediates SUMO conjugation at DNA lesions and facilitates DSB repair [221]
CHD1 [197]	Opens chromatin around DSBs to allow for recruitment of HR proteins [222]
DDB1 [23]	Part of UV damage recognition complex in NER [223]
DDB2 [197]	Part of UV damage recognition complex in NER [223]
DEK [23]	Structural modulator of chromatin [224]
PRKDC (aka DNA-PKcs) [23]	Canonical factor in DSB repair by NHEJ [137]
DYRK1A [197]	Regulates recruitment of 53BP1 to DNA damage sites, inhibiting NHEJ [225]
H2A1B [23]	Contributes to higher order chromatin structure [226,227,228]
HNRNPK [197]	Contributes to DNA damage signalling [229,230,231,232]
HNRNPU (aka SAF-A) [197]	Regulator of DNA-end resection [233]
LMNA [197]	Important for DSB repair and telomere maintenance [234,235]
PRDX1 [38,197]	Protects telomeres from oxidative damage [236]
RAN [197]	Regulates nuclear import of ATM [237,238]
RBBP4 [197]	As part of chromatin remodelling complexes regulates DNA repair [239]
RFC2 [23]	DNA replication factor involved in PCNA-related repair mechanisms [240]
SENP1 [197]	SUMO-deconjugating enzyme that regulates p53 activity [241]
SIN3A [197]	Restricts formation of potentially harmful R-loop structures in DNA [242]
SMAD4 [197]	Promotes expression of DSB and NER repair proteins [243]
SMARCE1 (aka SMCE1) [23]	Chromatin remodelling via SWI/SNF complex [244]
STK38 [199]	Facilitates cell cycle arrest [245] and promotes activation of ATM [246]
TOP2A [197]	Checkpoint for chromosome decatenation during mitosis [247]
UBE2C [88]	Regulator of cell cycle progression and arrest [248]
WDR33 [197]	Prevents genome instability caused by unreleased nascent transcripts [249]
XRCC5 (aka Ku80) [197]	Essential factor in DSB repair by NHEJ [250]
XRCC6 (aka Ku70) [88,197]	Essential factor in DSB repair by NHEJ [250]
ZNF281 [197]	Helps recruit XRCC4 to DNA breaks for DSB repair by NHEJ [251]

Abbreviations: BER: base excision repair; DSBs: DNA double-strand breaks; HR: homologous recombination; NER: nucleotide excision repair; NHEJ: non-homologous end-joining; PML: promyelocytic leukaemia; TLS: translesion DNA synthesis; UV light: ultraviolet light.

## References

[1] Cappadocia L., Lima C.D. (2018). Ubiquitin-like protein conjugation: Structures, chemistry, and mechanism. Chem. Rev..

[2] Hochstrasser M. (2009). Origin and function of ubiquitin-like proteins. Nature.

[3] Schwertman P., Bekker-Jensen S., Mailand N. (2016). Regulation of DNA double-strand break repair by ubiquitin and ubiquitin-like modifiers. Nat. Rev. Mol. Cell Biol..

[4] Hartmann-Petersen R., Gordon C. (2004). Integral UBL domain proteins: A family of proteasome interacting proteins. Semin. Cell Dev. Biol..

[5] Madsen L., Schulze A., Seeger M., Hartmann-Petersen R. (2007). Ubiquitin domain proteins in disease. BMC Biochem..

[6] Hoeller D., Hecker C.-M., Dikic I. (2006). Ubiquitin and ubiquitin-like proteins in cancer pathogenesis. Nat. Rev. Cancer.

[7] Kerscher O., Felberbaum R., Hochstrasser M. (2006). Modification of Proteins by Ubiquitin and Ubiquitin-Like Proteins. Annu. Rev. Cell Dev. Biol..

[8] Herrmann J., Lerman L.O., Lerman A. (2007). Ubiquitin and Ubiquitin-Like Proteins in Protein Regulation. Circ. Res..

[9] Osborne H.C., Irving E., Schmidt C.K. (2020). The Ubiquitin/UBL Drug Target Repertoire. Trends Mol. Med..

[10] Jackson S.P., Durocher D. (2013). Regulation of DNA damage responses by ubiquitin and SUMO. Mol. Cell.

[11] Brown J.S., Jackson S.P. (2015). Ubiquitylation, neddylation and the DNA damage response. Open Biol..

[12] Da Costa I.C., Schmidt C.K. (2020). Ubiquitin-like proteins in the DNA damage response: The next generation. Essays Biochem..

[13] Yu J., Qin B., Lou Z. (2020). Ubiquitin and ubiquitin-like molecules in DNA double strand break repair. Cell Biosci..

[14] Haas A.L., Ahrens P., Bright P.M., Ankel H. (1987). Interferon induced a 15-kilodalton protein exhibiting marked homology to ubiquitin. J. Biol. Chem..

[15] Perng Y.C., Lenschow D.J. (2018). ISG15 in antiviral immunity and beyond. Nat. Rev. Microbiol..

[16] Goldstein G., Scheid M., Hammerling U., Schlesinger D.H., Niall H.D., Boyse E.A. (1975). Isolation of a polypeptide that has lymphocyte differentiating properties and is probably represented universally in living cells. Proc. Natl. Acad. Sci. USA.

[17] Farrell P.J., Broeze R.J., Lengyel P. (1979). Accumulation of an mRNA and protein in interferon-treated Ehrlich ascites tumour cells. Nature.

[18] Loeb K.R., Haas A.L. (1992). The interferon-inducible 15-kDa ubiquitin homolog conjugates to intracellular proteins. J. Biol. Chem..

[19] D’Cunha J., Knight E., Haas A.L., Truitt R.L., Borden E.C. (1996). Immunoregulatory properties of ISG15, an interferon-induced cytokine. Proc. Natl. Acad. Sci. USA.

[20] Narasimhan J., Wang M., Fu Z., Klein J.M., Haas A.L., Kim J.-J.P. (2005). Crystal Structure of the Interferon-Induced Ubiquitin-Like Protein ISG15. J. Biol. Chem..

[21] Dao C.T., Zhang D.-E. (2005). ISG15: A ubiquitin-like enigma. Front. Biosci..

[22] Chang Y.G., Yan X.Z., Xie Y.Y., Gao X.C., Song A.X., Zhang D.E., Hu H.Y. (2008). Different roles for two ubiquitin-like domains of ISG15 in protein modification. J. Biol. Chem..

[23] Raso M.C., Djoric N., Walser F., Hess S., Schmid F.M., Burger S., Knobeloch K.-P., Penengo L. (2020). Interferon-stimulated gene 15 accelerates replication fork progression inducing chromosomal breakage. J. Cell Biol..

[24] Zhang D., Zhang D.E. (2011). Interferon-stimulated gene 15 and the protein ISGylation system. J. Interf. Cytokine Res..

[25] Magor K.E., Navarro D.M., Barber M.R.W., Petkau K., Fleming-Canepa X., Blyth G.A.D., Blaine A.H. (2013). Defense genes missing from the flight division. Dev. Comp. Immunol..

[26] Daczkowski C.M., Dzimianski J.V., Clasman J.R., Goodwin O., Mesecar A.D., Pegan S.D. (2017). Structural Insights into the Interaction of Coronavirus Papain-Like Proteases and Interferon-Stimulated Gene Product 15 from Different Species. J. Mol. Biol..

[27] Langley C., Goodwin O., Dzimianski J.V., Daczkowski C.M., Pegan S.D. (2019). Structure of interferon-stimulated gene product 15 (ISG15) from the bat species Myotis davidii and the impact of interdomain ISG15 interactions on viral protein engagement. Acta Crystallogr. Sect. D Struct. Biol..

[28] Jiang Y., Wang X. (2019). Structural insights into the species preference of the influenza B virus NS1 protein in ISG15 binding. Protein Cell.

[29] Zuin A., Isasa M., Crosas B. (2014). Ubiquitin Signaling: Extreme Conservation as a Source of Diversity. Cells.

[30] Knigth E., Fahey D., Cordova B., Hillman M., Kutny R., Reich N., Blomstrom D. (1988). A 15-kDa interferon-induced protein is derived by COOH-terminal processing of a 17-kDa precursor. J. Biol. Chem..

[31] Potter J.L., Narasimhan J., Mende-Mueller L., Haas A.L. (1999). Precursor processing of pro-ISG15/UCRP, an interferon-β-induced ubiquitin-like protein. J. Biol. Chem..

[32] Durfee L.A., Huibregtse J.M. (2012). The ISG15 Conjugation System. Methods Mol. Biol..

[33] Kim K., Giannakopoulos N.V., Virgin H.W., Zhang D.-E. (2004). Interferon-Inducible Ubiquitin E2, Ubc8, Is a Conjugating Enzyme for Protein ISGylation. Mol. Cell. Biol..

[34] Dastur A. (2006). Herc5, an Interferon-Induced HECT E3 Enzyme, Is Required for Conjugation of ISG15 in Human Cells. J. Biol. Chem..

[35] Zou W., Zhang D.-E. (2006). The interferon-inducible ubiquitin-protein isopeptide ligase (E3) EFP also functions as an ISG15 E3 ligase. J. Biol. Chem..

[36] Okumura F., Zou W., Zhang D.E. (2007). ISG15 modification of the eIF4E cognate 4EHP enhances cap structure-binding activity of 4EHP. Genes Dev..

[37] Kroismayr R., Baranyi U., Stehlik C., Dorfleutner A., Binder B.R., Lipp J. (2004). HERC5, a HECT E3 ubiquitin ligase tightly regulated in LPS activated endothelial cells. J. Cell Sci..

[38] Wong J.J.Y., Pung Y.F., Sze N.S.-K., Chin K.-C. (2006). HERC5 is an IFN-induced HECT-type E3 protein ligase that mediates type I IFN-induced ISGylation of protein targets. Proc. Natl. Acad. Sci. USA.

[39] Duda D.M., Olszewski J.L., Schuermann J.P., Kurinov I., Miller D.J., Nourse A., Alpi A.F., Schulman B.A. (2013). Structure of HHARI, a RING-IBR-RING ubiquitin ligase: Autoinhibition of an Ariadne-family E3 and insights into ligation mechanism. Structure.

[40] Martín-Vicente M., Medrano L.M., Resino S., García-Sastre A., Martínez I. (2017). TRIM25 in the regulation of the antiviral innate immunity. Front. Immunol..

[41] Zhao C., Beaudenon S.L., Kelley M.L., Waddell M.B., Yuan W., Schulman B.A., Huibregtse J.M., Krug R.M. (2004). The UbcH8 ubiquitin E2 enzyme is also the E2 enzyme for ISG15, an IFN-/-induced ubiquitin-like protein. Proc. Natl. Acad. Sci. USA.

[42] Durfee L.A., Kelley M.L., Huibregtse J.M. (2008). The basis for selective E1-E2 interactions in the ISG15 conjugation system. J. Biol. Chem..

[43] Malakhov M.P., Malakhova O.A., Kim K., Ritchie K.J., Zhang D.E. (2002). UBP43 (USP18) specifically removes ISG15 from conjugated proteins. J. Biol. Chem..

[44] Basters A., Geurink P.P., El Oualid F., Ketscher L., Casutt M.S., Krause E., Ovaa H., Knobeloch K.P., Fritz G. (2014). Molecular characterization of ubiquitin-specific protease 18 reveals substrate specificity for interferon-stimulated gene 15. FEBS J..

[45] Ritchie K.J., Hahn C.S., Kim K., Yan M., Rosario D., Li L., de la Torre J.C., Zhang D.-E. (2004). Role of ISG15 protease UBP43 (USP18) in innate immunity to viral infection. Nat. Med..

[46] Ketscher L., Hannß R., Morales D.J., Basters A., Guerra S., Goldmann T., Hausmann A., Prinz M., Naumann R., Pekosz A. (2015). Selective inactivation of USP18 isopeptidase activity in vivo enhances ISG15 conjugation and viral resistance. Proc. Natl. Acad. Sci. USA.

[47] Kao S.-Y. (2009). DNA damage induces nuclear translocation of parkin. J. Biomed. Sci..

[48] Kao S.-Y. (2009). Regulation of DNA repair by parkin. Biochem. Biophys. Res. Commun..

[49] Lee S.B., Kim J.J., Nam H.-J., Gao B., Yin P., Qin B., Yi S.-Y., Ham H., Evans D., Kim S.-H. (2015). Parkin Regulates Mitosis and Genomic Stability through Cdc20/Cdh1. Mol. Cell.

[50] Zhu X., Ma X., Tu Y., Huang M., Liu H., Wang F., Gong J., Wang J., Li X., Chen Q. (2017). Parkin regulates translesion DNA synthesis in response to UV radiation. Oncotarget.

[51] Zhang X.-W., Wang X.-F., Ni S.-J., Qin W., Zhao L.-Q., Hua R.-X., Lu Y.-W., Li J., Dimri G.P., Guo W.-J. (2015). UBTD1 induces cellular senescence through an UBTD1-Mdm2/p53 positive feedback loop. J. Pathol..

[52] Shkreta L., Chabot B. (2015). The RNA Splicing Response to DNA Damage. Biomolecules.

[53] Ng J.M.Y. (2003). A novel regulation mechanism of DNA repair by damage-induced and RAD23-dependent stabilization of xeroderma pigmentosum group C protein. Genes Dev..

[54] Castelli M., Pieroni S., Brunacci C., Piobbico D., Bartoli D., Bellet M.M., Colombo E., Pelicci P.G., Della Fazia M.A., Servillo G. (2013). Hepatocyte odd protein shuttling (HOPS) is a bridging protein in the nucleophosmin-p19Arf network. Oncogene.

[55] Fu H., Zhang Y., Chen J., Zhou B., Chen G., Chen P. (2020). Tmub1 Suppresses Hepatocellular Carcinoma by Promoting the Ubiquitination of ΔNp63 Isoforms. Mol. Ther. Oncolytics.

[56] Castelli M., Piobbico D., Chiacchiaretta M., Brunacci C., Pieroni S., Bartoli D., Gargaro M., Fallarino F., Puccetti P., Soddu S. (2020). HOPS/TMUB1 retains p53 in the cytoplasm and sustains p53-dependent mitochondrial apoptosis. EMBO Rep..

[57] Mistry H., Tamblyn L., Butt H., Sisgoreo D., Gracias A., Larin M., Gopalakrishnan K., Hande M., McPherson J. (2010). UHRF1 is a genome caretaker that facilitates the DNA damage response to γ-irradiation. Genome Integr..

[58] Chen H., Ma H., Inuzuka H., Diao J., Lan F., Shi Y.G., Wei W., Shi Y. (2013). DNA Damage Regulates UHRF1 Stability via the SCF β-TrCP E3 Ligase. Mol. Cell. Biol..

[59] Zhang H., Liu H., Chen Y., Yang X., Wang P., Liu T., Deng M., Qin B., Correia C., Lee S. (2016). A cell cycle-dependent BRCA1–UHRF1 cascade regulates DNA double-strand break repair pathway choice. Nat. Commun..

[60] Marteijn J.A., Lans H., Vermeulen W., Hoeijmakers J.H.J. (2014). Understanding nucleotide excision repair and its roles in cancer and ageing. Nat. Rev. Mol. Cell Biol..

[61] Uckelmann M., Densham R.M., Baas R., Winterwerp H.H.K., Fish A., Sixma T.K., Morris J.R. (2018). USP48 restrains resection by site-specific cleavage of the BRCA1 ubiquitin mark from H2A. Nat. Commun..

[62] Velimezi G., Robinson-Garcia L., Muñoz-Martínez F., Wiegant W.W., da Silva J.F., Owusu M., Moder M., Wiedner M., Rosenthal S.B., Fisch K.M. (2018). Map of synthetic rescue interactions for the Fanconi anemia DNA repair pathway identifies USP48. Nat. Commun..

[63] Lin T.-Y., Chan H.-H., Chen S.-H., Sarvagalla S., Chen P.-S., Coumar M.S., Cheng S.M., Chang Y.-C., Lin C.-H., Leung E. (2020). BIRC5/Survivin is a novel ATG12–ATG5 conjugate interactor and an autophagy-induced DNA damage suppressor in human cancer and mouse embryonic fibroblast cells. Autophagy.

[64] Krenciute G., Liu S., Yucer N., Shi Y., Ortiz P., Liu Q., Kim B.-J., Odejimi A.O., Leng M., Qin J. (2013). Nuclear BAG6-UBL4A-GET4 Complex Mediates DNA Damage Signaling and Cell Death. J. Biol. Chem..

[65] Yu S., Dai J., Ma M., Xu T., Kong Y., Cui C., Chi Z., Si L., Tang H., Yang L. (2019). RBCK1 promotes p53 degradation via ubiquitination in renal cell carcinoma. Cell Death Dis..

[66] Wang X.-H., O’Connor D., Brimmell M., Packham G. (2009). The BAG-1 cochaperone is a negative regulator of p73-dependent transcription. Br. J. Cancer.

[67] Zhang X.-Y., Pfeiffer H.K., Mellert H.S., Stanek T.J., Sussman R.T., Kumari A., Yu D., Rigoutsos I., Thomas-Tikhonenko A., Seidel H.E. (2011). Inhibition of the Single Downstream Target BAG1 Activates the Latent Apoptotic Potential of MYC. Mol. Cell. Biol..

[68] Paredes F., Parra V., Torrealba N., Navarro-Marquez M., Gatica D., Bravo-Sagua R., Troncoso R., Pennanen C., Quiroga C., Chiong M. (2016). HERPUD1 protects against oxidative stress-induced apoptosis through downregulation of the inositol 1,4,5-trisphosphate receptor. Free Radic. Biol. Med..

[69] Jachimowicz R.D., Beleggia F., Isensee J., Velpula B.B., Goergens J., Bustos M.A., Doll M.A., Shenoy A., Checa-Rodriguez C., Wiederstein J.L. (2019). UBQLN4 Represses Homologous Recombination and Is Overexpressed in Aggressive Tumors. Cell.

[70] Garvin A.J., Morris J.R. (2017). SUMO, a small, but powerful, regulator of double-strand break repair. Philos. Trans. R. Soc. B Biol. Sci..

[71] Dereeper A., Guignon V., Blanc G., Audic S., Buffet S., Chevenet F., Dufayard J.-F., Guindon S., Lefort V., Lescot M. (2008). Phylogeny.fr: Robust phylogenetic analysis for the non-specialist. Nucleic Acids Res..

[72] Letunic I., Bork P. (2019). Interactive Tree of Life (iTOL) v4: Recent updates and new developments. Nucleic Acids Res..

[73] Holst M., Saied F. (1993). Multigrid solution of the Poisson-Boltzmann equation. J. Comput. Chem..

[74] Holst M.J., Saied F. (1995). Numerical solution of the nonlinear Poisson-Boltzmann equation: Developing more robust and efficient methods. J. Comput. Chem..

[75] Holst M. (2001). Adaptive numerical treatment of elliptic systems on manifolds. Adv. Comput. Math..

[76] Baker N.A., Sept D., Joseph S., Holst M.J., McCammon J.A. (2001). Electrostatics of nanosystems: Application to microtubules and the ribosome. Proc. Natl. Acad. Sci. USA.

[77] Bank R.E., Holst M. (2003). A New Paradigm for Parallel Adaptive Meshing Algorithms. SIAM Rev..

[78] Jurrus E., Engel D., Star K., Monson K., Brandi J., Felberg L.E., Brookes D.H., Wilson L., Chen J., Liles K. (2018). Improvements to the APBS biomolecular solvation software suite. Protein Sci..

[79] Madeira F., Park Y.M., Lee J., Buso N., Gur T., Madhusoodanan N., Basutkar P., Tivey A.R.N., Potter S.C., Finn R.D. (2019). The EMBL-EBI search and sequence analysis tools APIs in 2019. Nucleic Acids Res..

[80] Robert X., Gouet P. (2014). Deciphering key features in protein structures with the new ENDscript server. Nucleic Acids Res..

[81] Malakhova O., Malakhov M., Hetherington C., Zhang D.E. (2002). Lipopolysaccharide activates the expression of ISG15-specific protease UBP43 via interferon regulatory factor 3. J. Biol. Chem..

[82] Pitha-Rowe I., Hasse B.A., Dmitrovsky E. (2004). Involvement of UBE1L in ISG15 Conjugation during Retinoid-induced Differentiation of Acute Promyelocytic Leukemia. J. Biol. Chem..

[83] Gentile M. (2003). Cell cycle arrest and apoptosis provoked by UV radiation-induced DNA damage are transcriptionally highly divergent responses. Nucleic Acids Res..

[84] Liu M., Hummer B.T., Li X., Hassel B.A. (2004). Camptothecin Induces the Ubiquitin-like Protein, ISG15, and Enhances ISG15 Conjugation in Response to Interferon. J. Interf. Cytokine Res..

[85] Jeon Y.J., Jo M.G., Yoo H.M., Hong S.H., Park J.M., Ka S.H., Oh K.H., Seol J.H., Jung Y.K., Chung C.H. (2012). Chemosensitivity is controlled by p63 modification with ubiquitin-like protein ISG15. J. Clin. Invest..

[86] Park J.M., Yang S.W., Yu K.R., Ka S.H., Lee S.W., Seol J.H., Jeon Y.J., Chung C.H. (2014). Modification of PCNA by ISG15 Plays a Crucial Role in Termination of Error-Prone Translesion DNA Synthesis. Mol. Cell.

[87] Jeon Y.J., Park J.H., Chung C.H. (2017). Interferon-Stimulated Gene 15 in the Control of Cellular Responses to Genotoxic Stress. Mol. Cells.

[88] Park J.H., Yang S.W., Park J.M., Ka S.H., Kim J.-H., Kong Y.-Y., Jeon Y.J., Seol J.H., Chung C.H. (2016). Positive feedback regulation of p53 transactivity by DNA damage-induced ISG15 modification. Nat. Commun..

[89] Radoshevich L., Impens F., Ribet D., Quereda J.J., Tham T.N., Nahori M.A., Bierne H., Dussurget O., Pizarro-Cerdá J., Knobeloch K.P. (2015). ISG15 counteracts Listeria monocytogenes infection. Elife.

[90] Lertsooksawat W., Wongnoppavich A., Chairatvit K. (2019). Up-regulation of interferon-stimulated gene 15 and its conjugation machinery, UbE1L and UbcH8 expression by tumor necrosis factor-α through p38 MAPK and JNK signaling pathways in human lung carcinoma. Mol. Cell. Biochem..

[91] Swaim C.D., Scott A.F., Canadeo L.A., Huibregtse J.M. (2017). Extracellular ISG15 Signals Cytokine Secretion through the LFA-1 Integrin Receptor. Mol. Cell.

[92] Dos Santos P.F., Mansur D.S. (2017). Beyond ISGlylation: Functions of Free Intracellular and Extracellular ISG15. J. Interf. Cytokine Res..

[93] Malakhova O.A., Kim K., Luo J.K., Zou W., Kumar K.G.S., Fuchs S.Y., Shuai K., Zhang D.E. (2006). UBP43 is a novel regulator of interferon signaling independent of its ISG15 isopeptidase activity. EMBO J..

[94] Liu M., Li X.-L., Hassel B.A. (2003). Proteasomes Modulate Conjugation to the Ubiquitin-like Protein, ISG15. J. Biol. Chem..

[95] Desai S.D., Haas A.L., Wood L.M., Tsai Y.C., Pestka S., Rubin E.H., Saleem A., Nur-E-Kamal A., Liu L.F. (2006). Elevated expression of ISG15 in tumor cells interferes with the ubiquitin/26S proteasome pathway. Cancer Res..

[96] Shi H.X., Yang K., Liu X., Liu X.Y., Wei B., Shan Y.F., Zhu L.H., Wang C. (2010). Positive Regulation of Interferon Regulatory Factor 3 Activation by Herc5 via ISG15 Modification. Mol. Cell. Biol..

[97] Wood L.M., Sankar S., Reed R.E., Haas A.L., Liu L.F., McKinnon P., Desai S.D. (2011). A Novel Role for ATM in Regulating Proteasome-Mediated Protein Degradation through Suppression of the ISG15 Conjugation Pathway. PLoS ONE.

[98] Ganesan M., Poluektova L.Y., Tuma D.J., Kharbanda K.K., Osna N.A. (2016). Acetaldehyde Disrupts Interferon Alpha Signaling in Hepatitis C Virus-Infected Liver Cells by Up-Regulating USP18. Alcohol. Clin. Exp. Res..

[99] Arimoto K.-I., Konishi H., Shimotohno K. (2008). UbcH8 regulates ubiquitin and ISG15 conjugation to RIG-I. Mol. Immunol..

[100] Li C., Wang J., Zhang H., Zhu M., Chen F., Hu Y., Liu H., Zhu H. (2014). Interferon-stimulated Gene 15 (ISG15) is a trigger for tumorigenesis and metastasis of hepatocellular carcinoma. Oncotarget.

[101] Fan J.-B., Arimoto K., Motamedchaboki K., Yan M., Wolf D.A., Zhang D.-E. (2015). Identification and characterization of a novel ISG15-ubiquitin mixed chain and its role in regulating protein homeostasis. Sci. Rep..

[102] Shah S.J., Blumen S., Pitha-Rowe I., Kitareewan S., Freemantle S.J., Feng Q., Dmitrovsky E. (2008). UBE1L represses PML/RARα by targeting the PML domain for ISG15ylation. Mol. Cancer Ther..

[103] Feng Q., Sekula D., Guo Y., Liu X., Black C.C., Galimberti F., Shah S.J., Sempere L.F., Memoli V., Andersen J.B. (2008). UBE1L causes lung cancer growth suppression by targeting cyclin D1. Mol. Cancer Ther..

[104] Huang Y.-F., Wee S., Gunaratne J., Lane D.P., Bulavin D.V. (2014). Isg15 controls p53 stability and functions. Cell Cycle.

[105] Park Y.S., Kwon Y.J., Chun Y.J. (2017). CYP1B1 activates Wnt/β-catenin signaling through suppression of Herc5-mediated ISGylation for protein degradation on β-catenin in HeLa cells. Toxicol. Res..

[106] Wang B., Li Y., Wang H., Zhao J., Zhao Y., Liu Z., Ma H. (2020). FOXO3a is stabilized by USP18-mediated de-ISGylation and inhibits TGF-β1-induced fibronectin expression. J. Investig. Med..

[107] Wan X., Chen H., Khan M.A., Xu A., Yang F., Zhang Y., Zhang D. (2013). ISG15 inhibits IFN-α-resistant liver cancer cell growth. Biomed Res. Int..

[108] Im E., Yoo L., Hyun M., Shin W.H., Chung K.C. (2016). Covalent ISG15 conjugation positively regulates the ubiquitin E3 ligase activity of parkin. Open Biol..

[109] Chen B., Retzlaff M., Roos T., Frydman J. (2011). Cellular strategies of protein quality control. Cold Spring Harb. Perspect. Biol..

[110] Yoo L., Yoon A.R., Yun C.O., Chung K.C. (2018). Covalent ISG15 conjugation to CHIP promotes its ubiquitin E3 ligase activity and inhibits lung cancer cell growth in response to type i interferon article. Cell Death Dis..

[111] Nakashima H., Nguyen T., Goins W.F., Chiocca E.A. (2015). Interferon-stimulated gene 15 (ISG15) and ISG15-linked proteins can associate with members of the selective autophagic process, histone deacetylase 6 (HDAC6) and SQSTM1/p62. J. Biol. Chem..

[112] Desai S.D., Reed R.E., Babu S., Lorio E.A. (2013). ISG15 deregulates autophagy in genotoxin-treated ataxia telangiectasia cells. J. Biol. Chem..

[113] Kim C.D., Reed R.E., Juncker M.A., Fang Z., Desai S.D. (2017). Evidence for the Deregulation of Protein Turnover Pathways in Atm-Deficient Mouse Cerebellum: An Organotypic Study. J. Neuropathol. Exp. Neurol..

[114] Falvey C.M., O’Donovan T.R., El-Mashed S., Nyhan M.J., O’Reilly S., McKenna S.L. (2017). UBE2L6/UBCH8 and ISG15 attenuate autophagy in esophageal cancer cells. Oncotarget.

[115] Li C., Wang Y., Zheng H., Dong W., Lv H., Lin J., Guo K., Zhang Y. (2020). Antiviral activity of ISG15 against classical swine fever virus replication in porcine alveolar macrophages via inhibition of autophagy by ISGylating BECN1. Vet. Res..

[116] Xu D., Zhang T., Xiao J., Zhu K., Wei R., Wu Z., Meng H., Li Y., Yuan J. (2015). Modification of BECN1 by ISG15 plays a crucial role in autophagy regulation by type I IFN/ interferon. Autophagy.

[117] Burks J., Reed R.E., Desai S.D. (2014). ISGylation governs the oncogenic function of Ki-Ras in breast cancer. Oncogene.

[118] Wang J.-M., Liu B.-Q., Zhang Q., Hao L., Li C., Yan J., Zhao F.-Y., Qiao H.-Y., Jiang J.-Y., Wang H.-Q. (2020). ISG15 suppresses translation of ABCC2 via ISGylation of hnRNPA2B1 and enhances drug sensitivity in cisplatin resistant ovarian cancer cells. Biochim. Biophys. Acta Mol. Cell Res..

[119] Okumura F., Okumura A.J., Uematsu K., Hatakeyama S., Zhang D.E., Kamura T. (2013). Activation of double-stranded rna-activated protein kinase (PKR) by interferon-stimulated gene 15 (ISG15) modification down-regulates protein translation. J. Biol. Chem..

[120] Holthaus D., Vasou A., Bamford C.G.G., Andrejeva J., Paulus C., Randall R.E., McLauchlan J., Hughes D.J. (2020). Direct Antiviral Activity of IFN-Stimulated Genes Is Responsible for Resistance to Paramyxoviruses in ISG15-Deficient Cells. J. Immunol..

[121] Durfee L.A., Lyon N., Seo K., Huibregtse J.M. (2010). The ISG15 Conjugation System Broadly Targets Newly Synthesized Proteins: Implications for the Antiviral Function of ISG15. Mol. Cell.

[122] Held T., Basler M., Knobeloch K., Groettrup M. (2020). Evidence for an involvement of the ubiquitin-like modifier ISG15 in MHC class I antigen presentation. Eur. J. Immunol..

[123] Villarroya-Beltri C., Baixauli F., Mittelbrunn M., Fernández-Delgado I., Torralba D., Moreno-Gonzalo O., Baldanta S., Enrich C., Guerra S., Sánchez-Madrid F. (2016). ISGylation controls exosome secretion by promoting lysosomal degradation of MVB proteins. Nat. Commun..

[124] Yeh Y.H., Yang Y.C., Hsieh M.Y., Yeh Y.C., Li T.K. (2013). A negative feedback of the HIF-1α pathway via interferon-stimulated gene 15 and ISGylation. Clin. Cancer Res..

[125] Fan J.-B., Miyauchi-Ishida S., Arimoto K., Liu D., Yan M., Liu C.-W., Győrffy B., Zhang D.-E. (2015). Type I IFN induces protein ISGylation to enhance cytokine expression and augments colonic inflammation. Proc. Natl. Acad. Sci. USA.

[126] Dos Santos P.F., Van Weyenbergh J., Delgobo M., de Patricio D.O., Ferguson B.J., Guabiraba R., Dierckx T., Menezes S.M., Báfica A., Mansur D.S. (2018). ISG15-Induced IL-10 Is a Novel Anti-Inflammatory Myeloid Axis Disrupted during Active Tuberculosis. J. Immunol..

[127] Østvik A.E., Svendsen T.D., van Beelen Granlund A., Doseth B., Skovdahl H.K., Bakke I., Thorsvik S., Afroz W., Walaas G.A., Mollnes T.E. (2020). Intestinal Epithelial Cells Express Immunomodulatory ISG15 during Active Ulcerative Colitis and Crohn’s Disease. J. Crohn’s Colitis.

[128] Swaim C.D., Canadeo L.A., Monte K.J., Khanna S., Lenschow D.J., Huibregtse J.M. (2020). Modulation of Extracellular ISG15 Signaling by Pathogens and Viral Effector Proteins. Cell Rep..

[129] Desai S.D., Reed R.E., Burks J., Wood L.M., Pullikuth A.K., Haas A.L., Liu L.F., Breslin J.W., Meiners S., Sankar S. (2012). ISG15 disrupts cytoskeletal architecture and promotes motility in human breast cancer cells. Exp. Biol. Med..

[130] Cerikan B., Shaheen R., Colo G.P., Gläßer C., Hata S., Knobeloch K.P., Alkuraya F.S., Fässler R., Schiebel E. (2016). Cell-Intrinsic Adaptation Arising from Chronic Ablation of a Key Rho GTPase Regulator. Dev. Cell.

[131] Hermann M., Bogunovic D. (2017). ISG15: In Sickness and in Health. Trends Immunol..

[132] Dzimianski J.V., Scholte F.E.M., Bergeron É., Pegan S.D. (2019). ISG15: It’s Complicated. J. Mol. Biol..

[133] Jackson S.P., Bartek J. (2009). The DNA-damage response in human biology and disease. Nature.

[134] Ciccia A., Elledge S.J. (2010). The DNA damage response: Making it safe to play with knives. Mol. Cell.

[135] Gaillard H., García-Muse T., Aguilera A. (2015). Replication stress and cancer. Nat. Rev. Cancer.

[136] Hanahan D., Weinberg R.A. (2011). Hallmarks of cancer: The next generation. Cell.

[137] Blackford A.N., Jackson S.P. (2017). ATM, ATR, and DNA-PK: The Trinity at the Heart of the DNA Damage Response. Mol. Cell.

[138] Hafner A., Bulyk M.L., Jambhekar A., Lahav G. (2019). The multiple mechanisms that regulate p53 activity and cell fate. Nat. Rev. Mol. Cell Biol..

[139] Gao Y., Mutter-Rottmayer E., Zlatanou A., Vaziri C., Yang Y. (2017). Mechanisms of Post-Replication DNA Repair. Genes.

[140] Sale J.E. (2013). Translesion DNA Synthesis and Mutagenesis in Eukaryotes. Cold Spring Harb. Perspect. Biol..

[141] Sharma S., Brosh R.M. (2007). Human RECQ1 Is a DNA Damage Responsive Protein Required for Genotoxic Stress Resistance and Suppression of Sister Chromatid Exchanges. PLoS ONE.

[142] Berti M., Chaudhuri A.R., Thangavel S., Gomathinayagam S., Kenig S., Vujanovic M., Odreman F., Glatter T., Graziano S., Mendoza-Maldonado R. (2013). Human RECQ1 promotes restart of replication forks reversed by DNA topoisomerase I inhibition. Nat. Struct. Mol. Biol..

[143] Reinhardt H.C., Yaffe M.B. (2013). Phospho-Ser/Thr-binding domains: Navigating the cell cycle and DNA damage response. Nat. Rev. Mol. Cell Biol..

[144] Huang Y.F., Bulavin D.V. (2014). Oncogene-mediated regulation of p53 ISGylation and functions. Oncotarget.

[145] Haupt Y., Maya R., Kazaz A., Oren M. (1997). Mdm2 promotes the rapid degradation of p53. Nature.

[146] Kubbutat M.H.G., Jones S.N., Vousden K.H. (1997). Regulation of p53 stability by Mdm2. Nature.

[147] Honda R., Tanaka H., Yasuda H. (1997). Oncoprotein MDM2 is a ubiquitin ligase E3 for tumor suppressor p53. FEBS Lett..

[148] Mercer W.E., Wiman K.G., Kohn K.W., Pietenpol J.A., Kastan M.B., Kinzler K.W., Vogelstein B. (1994). WAF1/CIP1 Is Induced in p53-mediated G1 Arrest and Apoptosis. Cancer Res..

[149] Toshiyuki M., Reed J.C. (1995). Tumor suppressor p53 is a direct transcriptional activator of the human bax gene. Cell.

[150] Nakano K., Vousden K.H. (2001). PUMA, a Novel Proapoptotic Gene, Is Induced by p53. Mol. Cell.

[151] Hummer B.T., Li X.-L., Hassel B.A. (2001). Role for p53 in Gene Induction by Double-Stranded RNA. J. Virol..

[152] Huo Y., Zong Z., Wang Q., Zhang Z., Deng H. (2017). ISG15 silencing increases cisplatin resistance via activating p53-mediated cell DNA repair. Oncotarget.

[153] Kuffour E.O., König R., Häussinger D., Schulz W.A., Münk C. (2019). ISG15 Deficiency Enhances HIV-1 Infection by Accumulating Misfolded p53. MBio.

[154] Forys J.T., Kuzmicki C.E., Saporita A.J., Winkeler C.L., Maggi L.B., Weber J.D. (2014). ARF and p53 Coordinate Tumor Suppression of an Oncogenic IFN-β-STAT1-ISG15 Signaling Axis. Cell Rep..

[155] Brown A.R., Simmen R.C.M., Raj V.R., Van T.T., MacLeod S.L., Simmen F.A. (2015). Krüppel-like factor 9 (KLF9) prevents colorectal cancer through inhibition of interferon-related signaling. Carcinogenesis.

[156] Tao J., Hua P., Wen J., Hu Y., Yang H., Xie X. (2015). Prognostic value of ISG15 mRNA level in drinkers with esophageal squamous cell cancers. Int. J. Clin. Exp. Pathol..

[157] Qiu X., Hong Y., Yang D., Xia M., Zhu H., Li Q., Xie H., Wu Q., Liu C., Zuo C. (2015). ISG15 as a novel prognostic biomarker for hepatitis B virus-related hepatocellular carcinoma. Int. J. Clin. Exp. Med..

[158] Hollingsworth J., Lau A., Tone A., Kollara A., Allen L., Colgan T.J., Dube V., Rosen B., Murphy K.J., Greenblatt E.M. (2018). BRCA1 Mutation Status and Follicular Fluid Exposure Alters NFκB Signaling and ISGylation in Human Fallopian Tube Epithelial Cells. Neoplasia.

[159] Andersen J.B., Aaboe M., Borden E.C., Goloubeva O.G., Hassel B.A., Ørntoft T.F. (2006). Stage-associated overexpression of the ubiquitin-like protein, ISG15, in bladder cancer. Br. J. Cancer.

[160] Bektas N., Noetzel E., Veeck J., Press M.F., Kristiansen G., Naami A., Hartmann A., Dimmler A., Beckmann M.W., Knüchel R. (2008). The ubiquitin-like molecule interferon-stimulated gene 15 (ISG15) is a potential prognostic marker in human breast cancer. Breast Cancer Res..

[161] Ina S., Hirono S., Noda T., Yamaue H. (2010). Identifying molecular markers for chemosensitivity to gemcitabine in pancreatic cancer: Increased expression of interferon-stimulated gene 15 kd is associated with intrinsic chemoresistance. Pancreas.

[162] Jinawath N., Furukawa Y., Hasegawa S., Li M., Tsunoda T., Satoh S., Yamaguchi T., Imamura H., Inoue M., Shiozaki H. (2004). Comparison of gene-expression profiles between diffuse- and intestinal-type gastric cancers using a genome-wide cDNA microarray. Oncogene.

[163] Laljee R.P., Muddaiah S., Salagundi B., Cariappa P.M., Indra A.S., Sanjay V., Ramanathan A. (2013). Interferon Stimulated Gene—ISG15 Is a Potential Diagnostic Biomarker in Oral Squamous Cell Carcinomas. Asian Pacific J. Cancer Prev..

[164] Padovan E., Terracciano L., Certa U., Jacobs B., Reschner A., Bolli M., Spagnoli G.C., Borden E.C., Heberer M. (2002). Interferon stimulated gene 15 constitutively produced by melanoma cells induces e-cadherin expression on human dendritic cells. Cancer Res..

[165] Talvinen K., Tuikkala J., Grönroos J., Huhtinen H., Kronqvist P., Aittokallio T., Nevalainen O., Hiekkanen H., Nevalainen T., Sundström J. (2006). Biochemical and clinical approaches in evaluating the prognosis of colon cancer. Anticancer Res..

[166] Wei J., Zaika E., Zaika A. (2012). p53 Family: Role of Protein Isoforms in Human Cancer. J. Nucleic Acids.

[167] Candi E., Dinsdale D., Rufini A., Salomoni P., Knight R.A., Mueller M., Krammer P.H., Melino G. (2007). TAp63 and ΔNp63 in Cancer and Epidermal Development. Cell Cycle.

[168] Ghosal G., Chen J. (2013). DNA damage tolerance: A double-edged sword guarding the genome. Transl. Cancer Res..

[169] Hoege C., Pfander B., Moldovan G.-L., Pyrowolakis G., Jentsch S. (2002). RAD6-dependent DNA repair is linked to modification of PCNA by ubiquitin and SUMO. Nature.

[170] Sale J.E., Lehmann A.R., Woodgate R. (2012). Y-family DNA polymerases and their role in tolerance of cellular DNA damage. Nat. Rev. Mol. Cell Biol..

[171] Mailand N., Gibbs-Seymour I., Bekker-Jensen S. (2013). Regulation of PCNA–protein interactions for genome stability. Nat. Rev. Mol. Cell Biol..

[172] Quinet A., Lemaçon D., Vindigni A. (2017). Replication Fork Reversal: Players and Guardians. Mol. Cell.

[173] Chen H., Chen H., Zhang J., Wang Y., Simoneau A., Yang H., Levine A.S., Zou L., Chen Z., Lan L. (2020). cGAS suppresses genomic instability as a decelerator of replication forks. Sci. Adv..

[174] Mosbech A., Gibbs-Seymour I., Kagias K., Thorslund T., Beli P., Povlsen L., Nielsen S.V., Smedegaard S., Sedgwick G., Lukas C. (2012). DVC1 (C1orf124) is a DNA damage-targeting p97 adaptor that promotes ubiquitin-dependent responses to replication blocks. Nat. Struct. Mol. Biol..

[175] Povlsen L.K., Beli P., Wagner S.A., Poulsen S.L., Sylvestersen K.B., Poulsen J.W., Nielsen M.L., Bekker-Jensen S., Mailand N., Choudhary C. (2012). Systems-wide analysis of ubiquitylation dynamics reveals a key role for PAF15 ubiquitylation in DNA-damage bypass. Nat. Cell Biol..

[176] Zeman M.K., Cimprich K.A. (2014). Causes and consequences of replication stress. Nat. Cell Biol..

[177] Meroni A., Vindigni A. (2020). ISG15 fast-tracks DNA replication. J. Cell Biol..

[178] Maya-Mendoza A., Moudry P., Merchut-Maya J.M., Lee M., Strauss R., Bartek J. (2018). High speed of fork progression induces DNA replication stress and genomic instability. Nature.

[179] Desai S.D. (2015). ISG15: A double edged sword in cancer. Oncoimmunology.

[180] Chun J.H., Kim H.K., Kim E., Kim I.H., Kim J.H., Chang H.J., Choi I.J., Lim H.S., Kim I.J., Kang H.C. (2004). Increased expression of metallothionein is associated with irinotecan resistance in gastric cancer. Cancer Res..

[181] Desai S.D., Wood L.M., Tsai Y.C., Hsieh T.S., Marks J.R., Scott G.L., Giovanella B.C., Liu L.F. (2008). ISG15 as a novel tumor biomarker for drug sensitivity. Mol. Cancer Ther..

[182] Tessema M., Yingling C.M., Thomas C.L., Klinge D.M., Bernauer A.M., Liu Y., Dacic S., Siegfried J.M., Dahlberg S.E., Schiller J.H. (2012). SULF2 methylation is prognostic for lung cancer survival and increases sensitivity to topoisomerase-I inhibitors via induction of ISG15. Oncogene.

[183] Shen J., Wei J., Wang H., Yue G., Yu L., Yang Y., Xie L., Zou Z., Qian X., Ding Y. (2013). A three-gene signature as potential predictive biomarker for irinotecan sensitivity in gastric cancer. J. Transl. Med..

[184] Luszczek W., Cheriyath V., Mekhail T.M., Borden E.C. (2010). Combinations of DNA methyltransferase and histone deacetylase inhibitors induce DNA damage in small cell lung cancer cells: Correlation of resistance with IFN-stimulated gene expression. Mol. Cancer Ther..

[185] Weichselbaum R.R., Ishwaran H., Yoon T., Nuyten D.S.A., Baker S.W., Khodarev N., Su A.W., Shaikh A.Y., Roach P., Kreike B. (2008). An interferon-related gene signature for DNA damage resistance is a predictive marker for chemotherapy and radiation for breast cancer. Proc. Natl. Acad. Sci. USA.

[186] Wilkins A.C., Patin E.C., Harrington K.J., Melcher A.A. (2019). The immunological consequences of radiation-induced DNA damage. J. Pathol..

[187] Erdal E., Haider S., Rehwinkel J., Harris A.L., McHugh P.J. (2017). A prosurvival DNA damage-induced cytoplasmic interferon response is mediated by end resection factors and is limited by Trex1. Genes Dev..

[188] Jacquelot N., Yamazaki T., Roberti M.P., Duong C.P.M., Andrews M.C., Verlingue L., Ferrere G., Becharef S., Vétizou M., Daillère R. (2019). Sustained type I interferon signaling as a mechanism of resistance to PD-1 blockade. Cell Res..

[189] Robin J.D., Ludlow A.T., Batten K., Magdinier F., Stadler G., Wagner K.R., Shay J.W., Wright W.E. (2014). Telomere position effect: Regulation of gene expression with progressive telomere shortening over long distances. Genes Dev..

[190] Lou Z., Wei J., Riethman H., Baur J.A., Voglauer R., Shay J.W., Wright W.E. (2009). Telomere length regulates ISG15 expression in human cells. Aging.

[191] Hirashima K., Migita T., Sato S., Muramatsu Y., Ishikawa Y., Seimiya H. (2013). Telomere Length Influences Cancer Cell Differentiation In Vivo. Mol. Cell. Biol..

[192] Hirashima K., Seimiya H. (2015). Telomeric repeat-containing RNA/G-quadruplex-forming sequences cause genome-wide alteration of gene expression in human cancer cells in vivo. Nucleic Acids Res..

[193] Mukherjee A.K., Sharma S., Sengupta S., Saha D., Kumar P., Hussain T., Srivastava V., Roy S.D., Shay J.W., Chowdhury S. (2018). Telomere length-dependent transcription and epigenetic modifications in promoters remote from telomere ends. PLoS Genet..

[194] Cong X., Yan M., Yin X., Zhang D. (2010). Hematopoietic cells from Ube1L-deficient mice exhibit an impaired proliferation defect under the stress of bone marrow transplantation. Blood Cells Mol. Dis..

[195] Vuillier F., Li Z., Commere P.H., Dynesen L.T., Pellegrini S. (2019). USP18 and ISG15 coordinately impact on SKP2 and cell cycle progression. Sci. Rep..

[196] Cai J., Liu T., Jiang X., Guo C., Liu A., Xiao X. (2017). Downregulation of USP18 inhibits growth and induces apoptosis in hepatitis B virus-related hepatocellular carcinoma cells by suppressing BCL2L1. Exp. Cell Res..

[197] Zhao C., Denison C., Huibregtse J.M., Gygi S., Krug R.M. (2005). Human ISG15 conjugation targets both IFN-induced and constitutively expressed proteins functioning in diverse cellular pathways. Proc. Natl. Acad. Sci. USA.

[198] Giannakopoulos N.V., Luo J.-K., Papov V., Zou W., Lenschow D.J., Jacobs B.S., Borden E.C., Li J., Virgin H.W., Zhang D.-E. (2005). Proteomic identification of proteins conjugated to ISG15 in mouse and human cells. Biochem. Biophys. Res. Commun..

[199] Takeuchi T., Inoue S., Yokosawa H. (2006). Identification and Herc5-mediated ISGylation of novel target proteins. Biochem. Biophys. Res. Commun..

[200] Sameer A.S., Nissar S. (2018). XPD-the lynchpin of NER: Molecule, gene, polymorphisms, and role in colorectal carcinogenesis. Front. Mol. Biosci..

[201] Guo Y., Dolinko A.V., Chinyengetere F., Stanton B., Bomberger J.M., Demidenko E., Zhou D.C., Gallagher R., Ma T., Galimberti F. (2010). Blockade of the ubiquitin protease UBP43 destabilizes transcription factor PML/RARa and inhibits the growth of acute promyelocytic leukemia. Cancer Res..

[202] Dellaire G., Ching R.W., Ahmed K., Jalali F., Tse K.C.K., Bristow R.G., Bazett-Jones D.P. (2006). Promyelocytic leukemia nuclear bodies behave as DNA damage sensors whose response to DNA double-strand breaks is regulated by NBS1 and the kinases ATM, Chk2, and ATR. J. Cell Biol..

[203] Xu Z.-X., Timanova-Atanasova A., Zhao R.-X., Chang K.-S. (2003). PML Colocalizes with and Stabilizes the DNA Damage Response Protein TopBP1. Mol. Cell. Biol..

[204] Parsons J.L., Tait P.S., Finch D., Dianova I.I., Allinson S.L., Dianov G.L. (2008). CHIP-Mediated Degradation and DNA Damage-Dependent Stabilization Regulate Base Excision Repair Proteins. Mol. Cell.

[205] Biswas K., Sarkar S., Du K., Brautigan D.L., Abbas T., Larner J.M. (2017). The E3 Ligase CHIP Mediates p21 Degradation to Maintain Radioresistance. Mol. Cancer Res..

[206] Jirawatnotai S., Sittithumcharee G. (2016). Paradoxical roles of cyclin D1 in DNA stability. DNA Repair.

[207] Reinhardt H.C., Schumacher B. (2014). The p53 network: Cellular and systemic DNA damage responses in aging and cancer. Trends Genet..

[208] Boehm E., Gildenberg M., Washington M. (2016). The many roles of PCNA in eukaryotic DNA replication. Enzymes.

[209] Di Masi A., Cilli D., Berardinelli F., Talarico A., Pallavicini I., Pennisi R., Leone S., Antoccia A., Noguera N.I., Lo-Coco F. (2016). PML nuclear body disruption impairs DNA double-strand break sensing and repair in APL. Cell Death Dis..

[210] Mustachio L.M., Kawakami M., Lu Y., Rodriguez-Canales J., Mino B., Behrens C., Wistuba I., Bota-Rabassedas N., Yu J., Lee J.J. (2017). The ISG15-specific protease USP18 regulates stability of PTEN. Oncotarget.

[211] Ming M., He Y.-Y. (2012). PTEN in DNA damage repair. Cancer Lett..

[212] Banerjee T., Sommers J.A., Huang J., Seidman M.M., Brosh R.M. (2015). Catalytic strand separation by RECQ1 is required for RPA-mediated response to replication stress. Curr. Biol..

[213] Hodge C.D., Spyracopoulos L., Glover J.N.M. (2016). Ubc13: The Lys63 ubiquitin chain building machine. Oncotarget.

[214] Brinkmann K., Schell M., Hoppe T., Kashkar H. (2015). Regulation of the DNA damage response by ubiquitin conjugation. Front. Genet..

[215] Torrecilla I., Oehler J., Ramadan K. (2017). The role of ubiquitin-dependent segregase p97 (VCP or Cdc48) in chromatin dynamics after DNA double strand breaks. Philos. Trans. R. Soc. B Biol. Sci..

[216] Kuper J., Braun C., Elias A., Michels G., Sauer F., Schmitt D.R., Poterszman A., Egly J.M., Kisker C. (2014). In TFIIH, XPD Helicase Is Exclusively Devoted to DNA Repair. PLoS Biol..

[217] Stiff T., Shtivelman E., Jeggo P., Kysela B. (2004). AHNAK interacts with the DNA ligase IV-XRCC4 complex and stimulates DNA ligase IV-mediated double-stranded ligation. DNA Repair.

[218] Leung J.W.C., Makharashvili N., Agarwal P., Chiu L.-Y., Pourpre R., Cammarata M.B., Cannon J.R., Sherker A., Durocher D., Brodbelt J.S. (2017). ZMYM3 regulates BRCA1 localization at damaged chromatin to promote DNA repair. Genes Dev..

[219] Massey T.H., Jones L. (2018). The central role of DNA damage and repair in CAG repeat diseases. Dis. Model. Mech..

[220] Bártová E., Malyšková B., Komůrková D., Legartová S., Suchánková J., Krejčí J., Kozubek S. (2017). Function of heterochromatin protein 1 during DNA repair. Protoplasma.

[221] Ismail I.H., Gagné J.P., Caron M.C., McDonald D., Xu Z., Masson J.Y., Poirier G.G., Hendzel M.J. (2012). CBX4-mediated SUMO modification regulates BMI1 recruitment at sites of DNA damage. Nucleic Acids Res..

[222] Kari V., Mansour W.Y., Raul S.K., Baumgart S.J., Mund A., Grade M., Sirma H., Simon R., Will H., Dobbelstein M. (2018). Loss of CHD 1 causes DNA repair defects and enhances prostate cancer therapeutic responsiveness. EMBO Rep..

[223] Sugasawa K. (2019). Mechanism and Regulation of DNA Damage Recognition in Mammalian Nucleotide Excision Repair.

[224] Teng Y., Lang L., Jauregui C.E. (2018). The Complexity of DEK Signaling in Cancer Progression. Curr. Cancer Drug Targets.

[225] Menon V.R., Ananthapadmanabhan V., Swanson S., Saini S., Sesay F., Yakovlev V., Florens L., DeCaprio J.A., Washburn M.P., Dozmorov M. (2019). DYRK1A regulates the recruitment of 53BP1 to the sites of DNA damage in part through interaction with RNF169. Cell Cycle.

[226] Miller K.M., Jackson S.P. (2012). Histone marks: Repairing DNA breaks within the context of chromatin. Biochem. Soc. Trans..

[227] Stadler J., Richly H. (2017). Regulation of DNA Repair Mechanisms: How the Chromatin Environment Regulates the DNA Damage Response. Int. J. Mol. Sci..

[228] Peuscher M.H., Jacobs J.J.L. (2011). DNA-damage response and repair activities at uncapped telomeres depend on RNF8. Nat. Cell Biol..

[229] Moumen A., Masterson P., O’Connor M.J., Jackson S.P. (2005). hnRNP K: An HDM2 Target and Transcriptional Coactivator of p53 in Response to DNA Damage. Cell.

[230] Moumen A., Magill C., Dry K., Jackson S.P. (2013). ATM-dependent phosphorylation of heterogeneous nuclear ribonucleoprotein K promotes p53 transcriptional activation in response to DNA damage. Cell Cycle.

[231] Blasius M., Bartek J. (2013). ATM targets hnRNPK to control p53. Cell Cycle.

[232] Wiesmann N., Strozynski J., Beck C., Zimmermann N., Mendler S., Gieringer R., Schmidtmann I., Brieger J. (2017). Knockdown of hnRNPK leads to increased DNA damage after irradiation and reduces survival of tumor cells. Carcinogenesis.

[233] Polo S.E., Blackford A.N., Chapman J.R., Baskcomb L., Gravel S., Rusch A., Thomas A., Blundred R., Smith P., Kzhyshkowska J. (2012). Regulation of DNA-end resection by hnRNPU-like proteins promotes DNA double-strand break signaling and repair. Mol. Cell.

[234] Gonzalo S. (2014). DNA Damage and Lamins. Advances in Experimental Medicine and Biology.

[235] Li W., Bai X., Li J., Zhao Y., Liu J., Zhao H., Liu L., Ding M. (2019). The nucleoskeleton protein IFFO1 immobilizes broken DNA and suppresses chromosome translocation during tumorigenesis. Nature Cell Biol..

[236] Aeby E., Ahmed W., Redon S., Simanis V., Lingner J. (2016). Peroxiredoxin 1 Protects Telomeres from Oxidative Damage and Preserves Telomeric DNA for Extension by Telomerase. Cell Rep..

[237] Cekan P., Hasegawa K., Pan Y., Tubman E., Odde D., Chen J.-Q., Herrmann M.A., Kumar S., Kalab P. (2016). RCC1-dependent activation of Ran accelerates cell cycle and DNA repair, inhibiting DNA damage–induced cell senescence. Mol. Biol. Cell.

[238] Dworak N., Makosa D., Chatterjee M., Jividen K., Yang C.S., Snow C., Simke W.C., Johnson I.G., Kelley J.B., Paschal B.M. (2019). A nuclear lamina-chromatin-Ran GTPase axis modulates nuclear import and DNA damage signaling. Aging Cell.

[239] Kitange G.J., Mladek A.C., Schroeder M.A., Pokorny J.C., Carlson B.L., Zhang Y., Nair A.A., Lee J.H., Yan H., Decker P.A. (2016). Retinoblastoma Binding Protein 4 Modulates Temozolomide Sensitivity in Glioblastoma by Regulating DNA Repair Proteins. Cell Rep..

[240] Li Y., Gan S., Ren L., Yuan L., Liu J., Wang W., Wang X., Zhang Y., Jiang J., Zhang F. (2018). Multifaceted regulation and functions of replication factor C family in human cancers. Am. J. Cancer Res..

[241] Dou H., Huang C., Singh M., Carpenter P.B., Yeh E.T.H. (2010). Regulation of DNA repair through deSUMOylation and SUMOylation of replication protein a complex. Mol. Cell.

[242] Salas-Armenteros I., Pérez-Calero C., Bayona-Feliu A., Tumini E., Luna R., Aguilera A. (2017). Human THO–Sin3A interaction reveals new mechanisms to prevent R-loops that cause genome instability. EMBO J..

[243] Zhao M., Mishra L., Deng C.X. (2018). The role of TGF-β/SMAD4 signaling in cancer. Int. J. Biol. Sci..

[244] Ribeiro-Silva C., Vermeulen W., Lans H. (2019). SWI/SNF: Complex complexes in genome stability and cancer. DNA Repair.

[245] Fukasawa T., Enomoto A., Miyagawa K. (2015). Serine-Threonine Kinase 38 regulates CDC25A stability and the DNA damage-induced G2/M checkpoint. Cell. Signal..

[246] Qin B., Yu J., Nowsheen S., Zhao F., Wang L., Lou Z. (2020). STK38 promotes ATM activation by acting as a reader of histone H4 ufmylation. Sci. Adv..

[247] Chen T., Sun Y., Ji P., Kopetz S., Zhang W. (2015). Topoisomerase IIα in chromosome instability and personalized cancer therapy. Oncogene.

[248] Bajaj S., Alam S.K., Roy K.S., Datta A., Nath S., Roychoudhury S. (2016). E2 Ubiquitin-Conjugating Enzyme, UBE2C Gene, Is Reciprocally Regulated by Wild-type and Gain-of-Function Mutant p53. J. Biol. Chem..

[249] Teloni F., Michelena J., Lezaja A., Kilic S., Ambrosi C., Menon S., Dobrovolna J., Imhof R., Janscak P., Baubec T. (2019). Efficient Pre-mRNA Cleavage Prevents Replication-Stress-Associated Genome Instability. Mol. Cell.

[250] Chang H.H.Y., Pannunzio N.R., Adachi N., Lieber M.R. (2017). Non-homologous DNA end joining and alternative pathways to double-strand break repair. Nat. Rev. Mol. Cell Biol..

[251] Nicolai S., Mahen R., Raschellà G., Marini A., Pieraccioli M., Malewicz M., Venkitaraman A.R., Melino G. (2020). ZNF281 is recruited on DNA breaks to facilitate DNA repair by non-homologous end joining. Oncogene.

[252] Malakhova O.A., Zhang D.E. (2008). ISG15 inhibits Nedd4 ubiquitin E3 activity and enhances the innate antiviral response. J. Biol. Chem..

[253] Minakawa M., Sone T., Takeuchi T., Yokosawa H. (2008). Regulation of the nuclear factor (NF)-κB pathway by ISGylation. Biol. Pharm. Bull..

[254] Soria-Bretones I., Cepeda-García C., Checa-Rodriguez C., Heyer V., Reina-San-Martin B., Soutoglou E., Huertas P. (2017). DNA end resection requires constitutive sumoylation of CtIP by CBX4. Nat. Commun..

[255] Takeuchi T., Yokosawa H. (2005). ISG15 modification of Ubc13 suppresses its ubiquitin-conjugating activity. Biochem. Biophys. Res. Commun..

[256] Bade V.N., Nickels J., Keusekotten K., Praefcke G.J.K. (2012). Covalent protein modification with ISG15 via a conserved cysteine in the hinge region. PLoS ONE.

[257] Rhee H.-W., Zou P., Udeshi N.D., Martell J.D., Mootha V.K., Carr S.A., Ting A.Y. (2013). Proteomic mapping of mitochondria in living cells via spatially restricted enzymatic tagging. Science.

[258] Bakos G., Yu L., Gak I.A., Roumeliotis T.I., Liakopoulos D., Choudhary J.S., Mansfeld J. (2018). An E2-ubiquitin thioester-driven approach to identify substrates modified with ubiquitin and ubiquitin-like molecules. Nat. Commun..

[259] Gupta R., Somyajit K., Narita T., Maskey E., Stanlie A., Kremer M., Typas D., Lammers M., Mailand N., Nussenzweig A. (2018). DNA Repair Network Analysis Reveals Shieldin as a Key Regulator of NHEJ and PARP Inhibitor Sensitivity. Cell.

[260] Brüninghoff K., Aust A., Taupitz K.F., Wulff S., Dörner W., Mootz H.D. (2020). Identification of SUMO binding proteins enriched after covalent photo-crosslinking. ACS Chem. Biol..

[261] Li T., Chen Z.J. (2018). The cGAS-cGAMP-STI NG pathway connects DNA damage to inflammation, senescence, and cancer. J. Exp. Med..

[262] Neves-Costa A., Moita L.F. (2017). Modulation of inflammation and disease tolerance by DNA damage response pathways. FEBS J..

[263] Morales A.J., Carrero J.A., Hung P.J., Tubbs A.T., Andrews J.M., Edelson B.T., Calderon B., Innes C.L., Paules R.S., Payton J.E. (2017). A type I IFN-dependent DNA damage response regulates the genetic program and inflammasome activation in macrophages. Elife.

[264] Du Y., Duan T., Feng Y., Liu Q., Lin M., Cui J., Wang R. (2018). LRRC25 inhibits type I IFN signaling by targeting ISG15-associated RIG-I for autophagic degradation. EMBO J..

[265] Zhang X., Bogunovic D., Payelle-Brogard B., Francois-Newton V., Speer S.D., Yuan C., Volpi S., Li Z., Sanal O., Mansouri D. (2015). Human intracellular ISG15 prevents interferon-α/β over-amplification and auto-inflammation. Nature.

[266] Zhang Y., Thery F., Wu N.C., Luhmann E.K., Dussurget O., Foecke M., Bredow C., Jiménez-Fernández D., Leandro K., Beling A. (2019). The in vivo ISGylome links ISG15 to metabolic pathways and autophagy upon Listeria monocytogenes infection. Nat. Commun..

